# Synthetic Modifications of a Pb^2+^-Sensor Acridono-Crown Ether for Covalent Attachment and Their Effects on Selectivity

**DOI:** 10.3390/molecules29051121

**Published:** 2024-03-01

**Authors:** Panna Vezse, Martin Gede, Ádám Golcs, Péter Huszthy, Tünde Tóth

**Affiliations:** 1 Department of Organic Chemistry and Technology, Budapest University of Technology and Economics, Szent Gellért Sq. 4, H-1111 Budapest, Hungary; panna.vezse@edu.bme.hu (P.V.); martin.gede@kaust.edu.sa (M.G.); huszthy.peter@vbk.bme.hu (P.H.); 2 Advanced Membranes and Porous Materials Center, Physical Science and Engineering Division, King Abdullah University of Science and Technology, Thuwal 23955-6900, Saudi Arabia; 3 Department of Pharmaceutical Chemistry, Semmelweis University, Hőgyes Endre Str. 9, H-1092 Budapest, Hungary; 4 HUN-REN Centre for Energy Research, Konkoly-Thege Miklós Str. 29-33, H-1121 Budapest, Hungary

**Keywords:** acridone, acridine, lead, crown ethers, UV–Vis spectroscopy

## Abstract

Because of environmental impact, there is a great need for chemosensors, especially for toxic heavy metals such as lead. The conventional instrumental analytical techniques rarely provide an available real-time sensing platform, thus the development of highly selective and stable synthetic chemosensor molecules is of great importance. Acridono-18-crown-6 ethers have such properties, and much research has proven their outstanding applicability in various supramolecular devices. In this present work, we aimed to enable their covalent immobilization capability by synthesizing functionalized derivatives while preserving the favored molecular recognition ability. Several new macrocycle analogues were synthesized, while synthetization difficulties and design aspects were also dealt with. The selectivity of the macrocycle analogues was studied using UV–Vis spectroscopy and compared with that of the parent compounds. The ultimate crown ether derivative showed high Pb^2+^-selectivity, reversibility (decomplexation by extraction with water) and stability.

## 1. Introduction

Crown ethers are one of the first simple-structured synthetic host molecules able to selectively recognize guest molecules analogous to biological receptors. Molecular recognition of these first-generation host molecules has been well-described in the past few decades of supramolecular chemistry research [1]. By utilizing synthetic host molecules, diverse functions can be created for component-specific chemical analysis and separation technology, thus providing useful methods and tools for environmental protection, nanotechnology, pharmaceutical, food and other industries. Today, crown ethers have enabled the introduction of many commercially available products (e.g., CROWNPAK^®^ chiral HPLC columns [2]) and industrial applications (e.g., IBC Advanced Technologies^®^ [3]). One of the favorable features of crown ethers is the simplicity of their molecular structure. Although this structural simplicity does not allow for the highly selective recognition of organic molecules, a very high specificity can be achieved in the complexation of inorganic cations. Moreover, the robust character and stability of these host molecules highly surpass those of bioreceptors. In addition, crown ethers can also be synthesized by using relatively inexpensive starting materials, even in large-scale industrial situations, which make them more readily available for practical applications [4,5,6].

However, current sustainability requirements encourage chemists to regenerate these host molecules, even if they are easily obtainable. In practice, this requires the covalent immobilization of the host molecules on various solid carriers, such as a stationary phase [7,8], a nanoparticle [9], a resin [10], a polymer [11], etc. Also, the chemical incorporation of the host molecules into solid carriers prevents the contamination of the samples with ionophores during analysis. Thus, crown compounds are functionalized with reactive groups. These are often inactive in molecular recognition, but provide additional functional groups for post-synthetic modifications and chemical immobilizations [5]. These structurally incorporated anchoring functions are especially advantageous if they show high versatility for synthetic modifications without influencing the coordination properties of the backbone of the host molecule. Achieving this kind of functionalization is one of the main goals of the present study.

In our research group, we were interested in analyzing Pb^2+^ using chemosensors. This research focuses on overcoming the main limitations of conventional chemosensing methods, with a strong emphasis on their availability and future adaptability. Consequently, inexpensive and simple-structured host molecules (e.g., crown ethers) with the possibility of covalent functionalization need to be used to satisfy these requirements. Of course, it is possible to replace chemosensing methods with instrumental analytical techniques, such as atomic absorption spectrometry (AAS), inductively coupled plasma techniques (ICP-MS and ICP-OES) or anodic stripping voltammetry (ASV). These methods allow for accurate and precise analyses of complex samples, but they are time-consuming and often require extensive sample preparation [12,13]. This makes them less suitable for rapid real-time analysis, high-throughput monitoring and the development of portability. Although they play an important role in modern analytical chemistry, there is a need for new chemosensing devices that can even be used by non-professionals, especially in developing countries.

Among the newly reported sensor molecules, Pb^2+^-selective ones have always been a focus of interest due to the high contamination and toxicity of lead. It can enter the human body by inhalation (from lead-containing fuel vapors) and through the gastrointestinal tract (from lead-containing food or drinking water), where the absorbed lead accumulates mainly in soft tissues and bones [14,15]. Moreover, Pb^2+^ can easily attach to the thiol groups of proteins [16], phosphates and sulfhydryl groups [17]. Furthermore, it can bind to various enzymes, changing their structure and reducing their activity.

**Figure 1:**
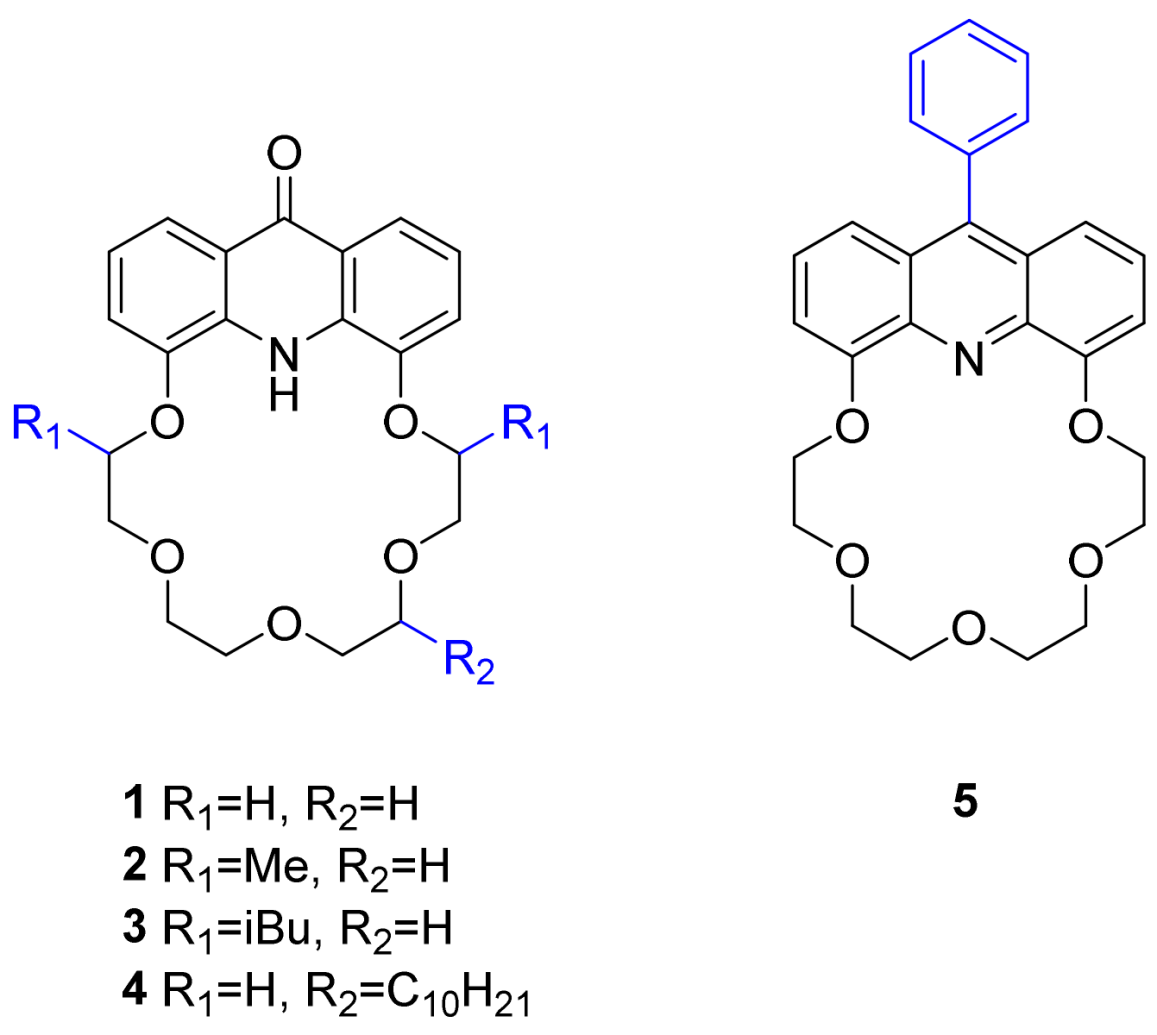
The proposed acridono-18-crown-6 ether macrocycle backbone **1** [18] for obtaining functionalizable and versatile Pb^2+^-selective ionophores and the previously reported Pb^2+^-complexing analogues **2**–**5** [19,20,21].

Ionophores with an acridono-18-crown-6 ether macrocycle backbone (see Figure 1) were often proposed for applications such as Pb^2+^-selective selectors and chemosensing situations.

The properties, i.e., selectivity, pH-sensitivity, stability, reversibility, fluorescence properties, etc. of these host molecules have often surpassed those of other small-molecule-based synthetic receptors, such as oxa- or dioxadicarboxylic acid diamides, diphenyl methyl-*N*-phenyl hydroxamic acid, antipyryliminomethinylphenyl-, quinoline-, bis(dithiocarbamate)-, rhodamine-B- and thiourea derivatives [22,23,24]. The superior selectivity is attributed to the multiple-binding inclusion complexation and neutrality over the simpler, functional group- or charge-driven intermolecular coordination, while the improved pH sensitivity is due to the basicity decrease in the acridine-*N* upon complexation.

**Figure 2:**
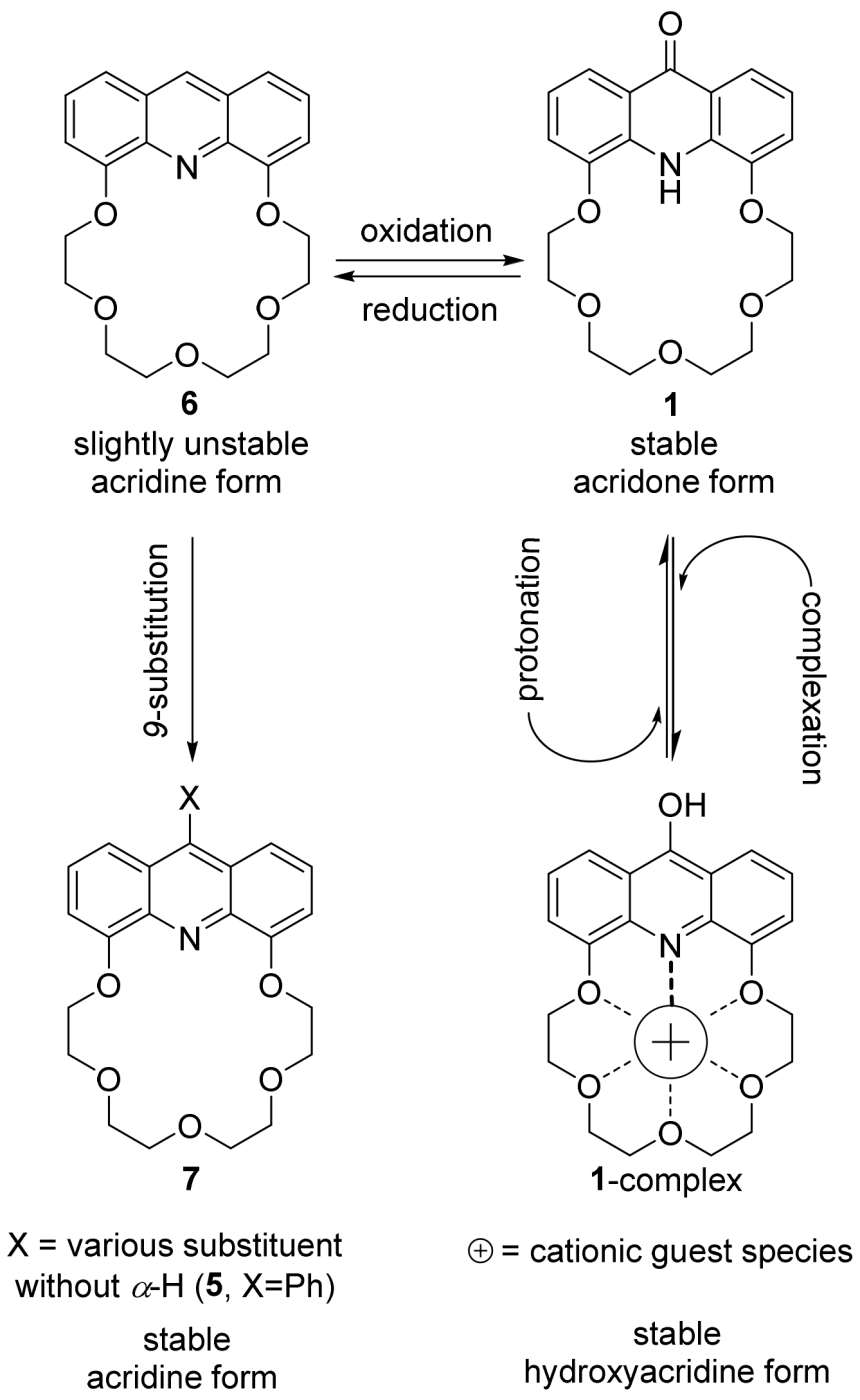
Typical structural transformations during practical applications of crown ether-type host molecules containing an acridone (**1**) or an acridine (**5**–**7**) unit on the examples of achiral analogues (the curved arrows represent the equilibrium shifting effects).

The most important structural transformations and equilibrium processes of this group of ionophores are summarized in Figure 2.

**Figure 3:**
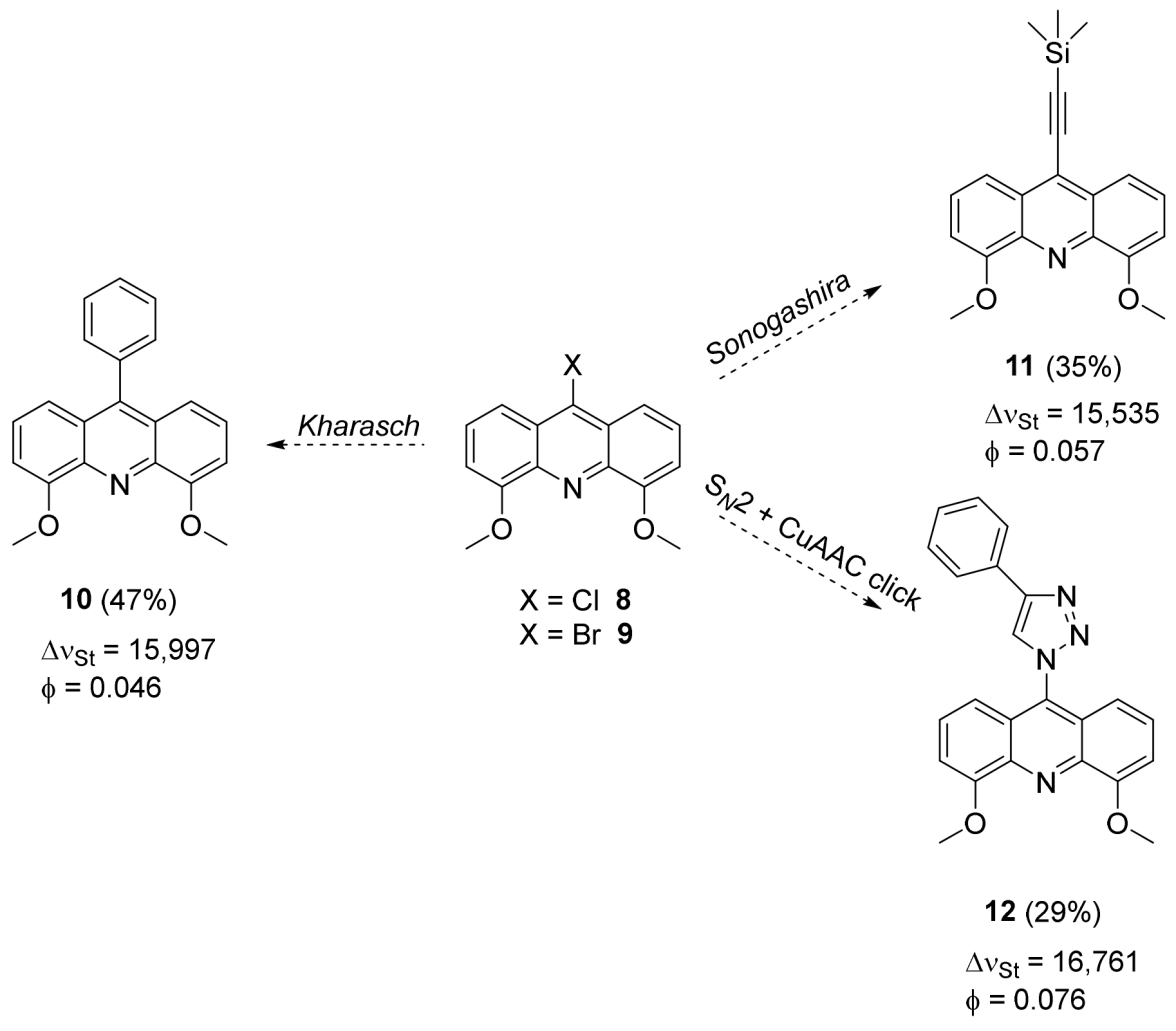
Promising methods for substitutions at position *9* of the acridine unit after transformation of the acridone moiety of the Pb^2+^-selective parent acridono-crown ether (**1**) into haloacridines (**8** and **9**, [25]) and the advantageous photophysical properties (Δν_St_ refers to the *Stokes*-shift in cm^−1^, while ϕ is the fluorescence quantum yield) of the resulting precursors (**10**–**12**, [25]).

Parent crown ethers or their corresponding heteroaromatic precursors can effectively be modified by taking two reported routes [25]. In the first approach, the acridine unit can be substituted at position *9* via a haloacridine derivative by using a *Kharasch*-type cross-coupling reaction and *Grignard*-reagents to introduce electron-donating groups (e.g., **5** in Figure 1), while electron-withdrawing groups can be introduced using a *Sonogashira* or click reaction (Figure 3). Naturally, there are several other synthetic possibilities as well, but the reported ones are the best in terms of the yield, mild reaction conditions, future applicability and fluorescence properties of the previously investigated precursors and structural analogues [25].

The other possibility is incorporating substituents into the oligoether unit of the crown ethers (e.g., **2**–**4** in Figure 1). This approach requires a longer synthetic route, as the additional functional groups have to be introduced during the synthesis of the oligoether precursors because the oligoether units of the crown ethers do not have the appropriate reactivity.

We report herein studies on the functionalizability of acridino-crown ethers to obtain covalently immobilizable analogues for the future development of supramolecular devices while aiming to preserve the high Pb^2+^-selectivity of the acridono-18-crown-6 backbone (**1**).

## 2. Results and Discussion

### 2.1. Structure Design

**Figure 4:**
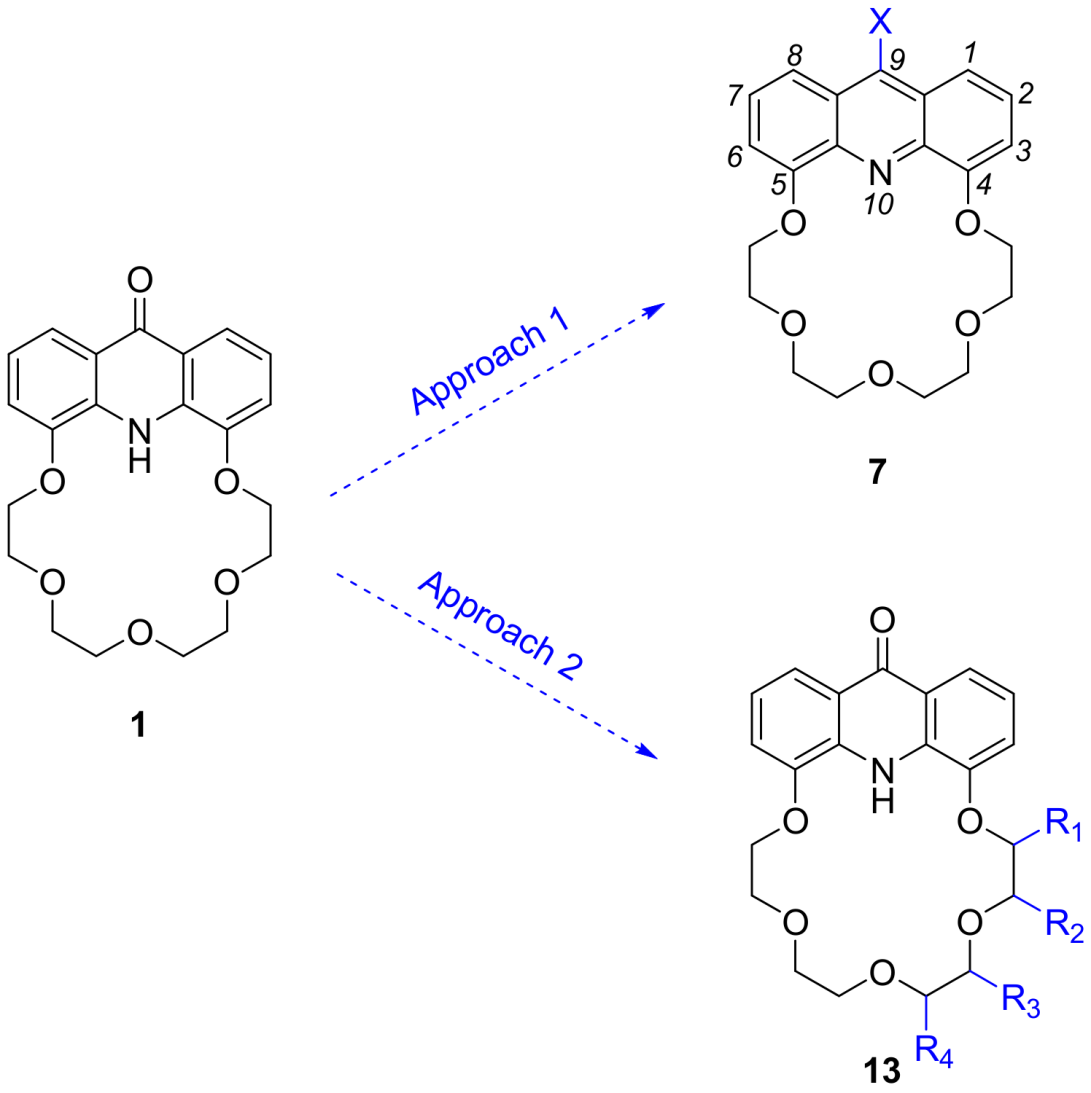
The applied synthetic approaches to obtain the desired covalently immobilizable Pb^2+^-selective crown ethers (**7**,**13**).

The functionalization of the acridono-crown ether backbone was carried out by using two different approaches (Figure 4), including the post-synthetic modification of the acridone unit and the introduction of reactive groups into the oligoether unit during the synthesis of the corresponding precursor.

The newly incorporated functions have been designed to enable easy and simple activation under mild reaction conditions for the subsequent covalent attachment of the host molecules. Thus, diethyl-acetal, methyl ester and benzyloxy functions were chosen for this purpose. Another important aspect is that the desired crown ethers should also contain a molecular spacer between the coordination sphere and the functionalizable group. This should generally be of a minimum 3 *C*-*C*-bond-lengths for maintaining the effective molecular recognition properties of the receptor unit.

### 2.2. Functionalization via 9-Substitution of the Acridine Unit (Approach 1)

Initially, the modification of the acridone unit of the host molecule was attempted by introducing a functionalizable group to position *9*. The desired crown ether contains a phenyl group functionalized at the *para*-position and was prepared via a multistep reaction pathway involving a *Kharasch*-coupling of a haloacridino-crown ether and a *Grignard*-reagent.

**Scheme 1:**
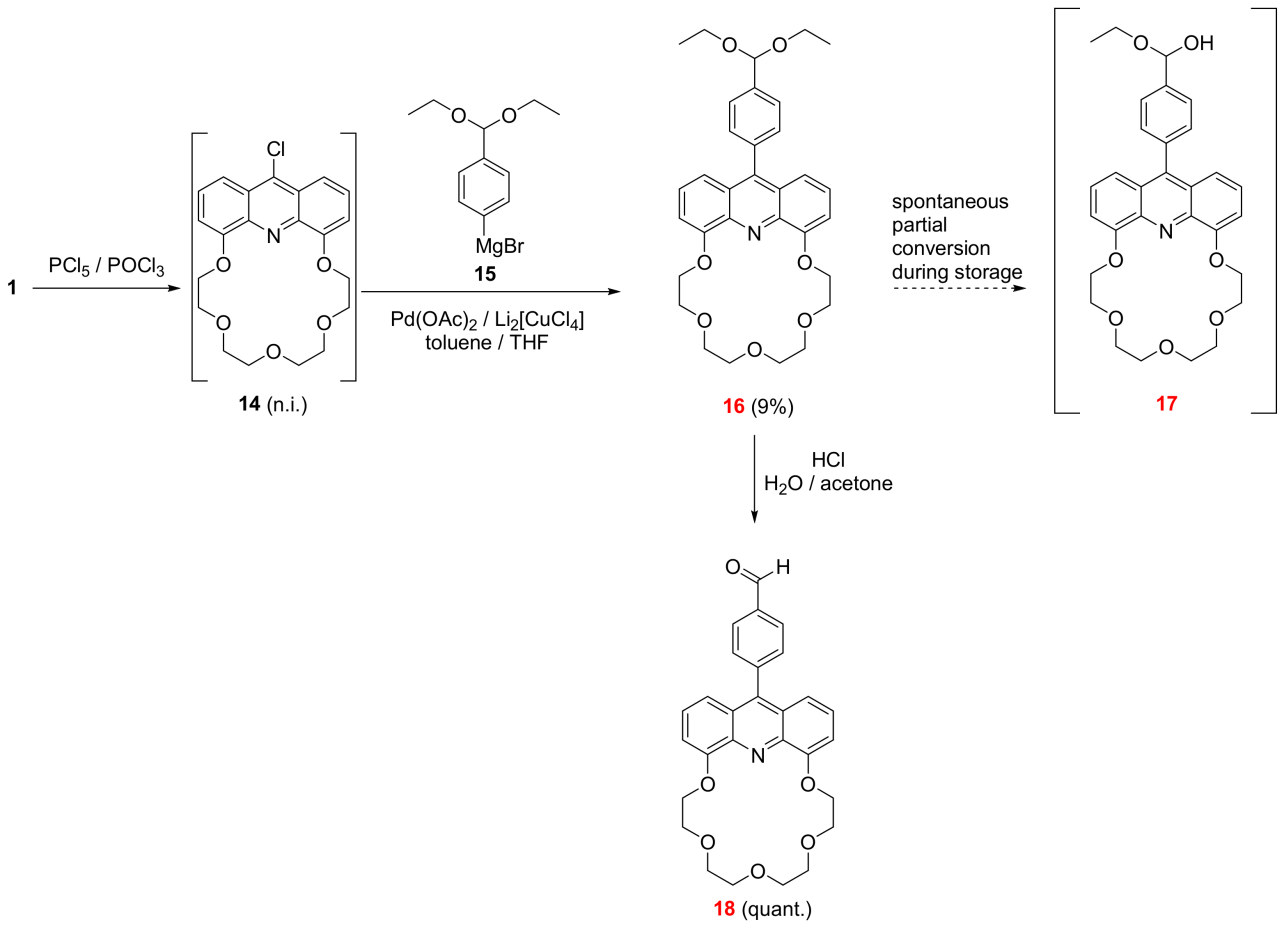
Synthesis of the aldehyde-moiety-containing crown ether analogue **18** by introducing a functionalizable unit at position *9* of acridine (the new compounds are indicated with numbers in red, while ‘n.i.’ means ‘not isolated’).

The starting material was the parent acridono-18-crown-6 ether (**1**), which was transformed into a highly reactive, but sufficiently stable 9-chloroacridino-crown ether (more stable than the corresponding 9-bromo- or 9-iodo-analogues) using phosphorus oxychloride and phosphorus pentachloride (Scheme 1) following to a reported procedure [25]. This intermediate (**14**) was not isolated, but was reacted as a crude product due to its instability.

The other key intermediate **15** was obtained by converting *para*-bromo-benzaldehyde-diethyl-acetal into a *Grignard*-reagent [26]. Diacetal **15** was used in the next step without isolation. The key intermediates (**14**,**15**) were reacted in a *Kharasch*-coupling to result in crown ether **16** containing a formyl group protected as an acetal. This product (**16**) was unstable and partially decomposed to the corresponding hemiacetal (**17**) and formyl (**18**) derivatives spontaneously at room temperature and in air. Finally, the desired compound **18** was obtained with a quantitative yield by an acidic hydrolysis of diacetal **16**. Compound **18** can easily be modified further by converting its reactive formyl group to suitable ones for covalent attachment to several Supplementary Materials.

**Scheme 2:**
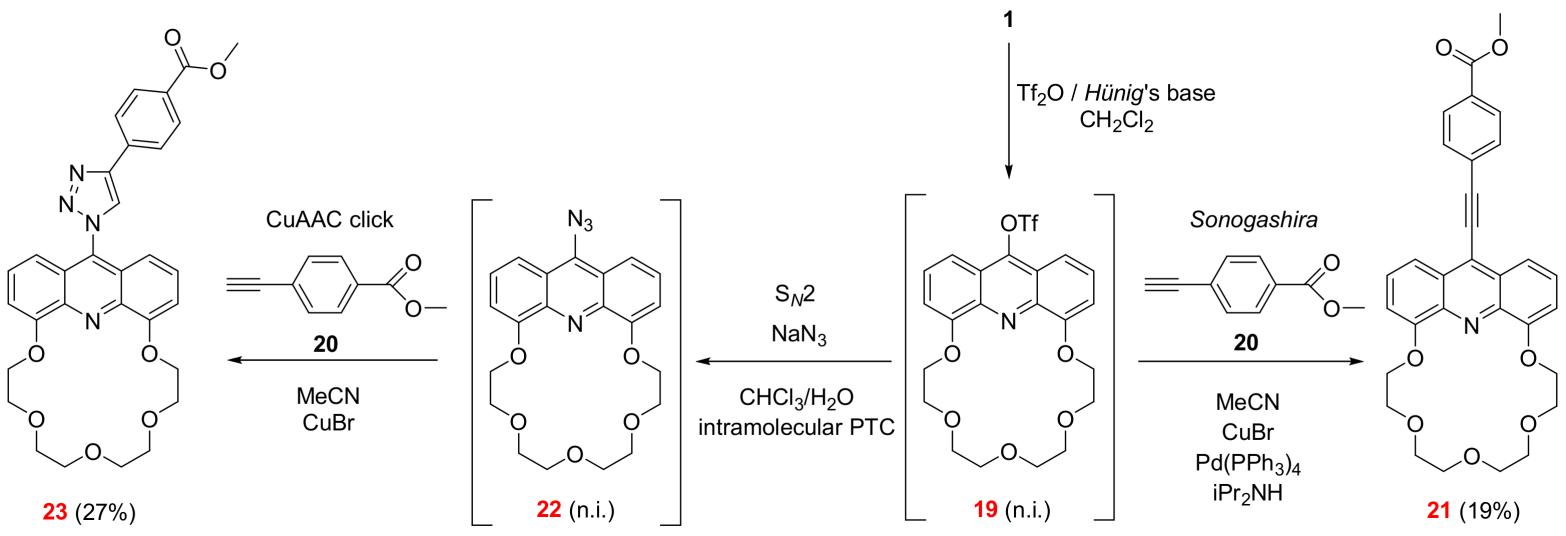
Introducing an easily functionalizable methyl ester unit by modification of the parent crown ether (**1**) via a triple-bond or a triazole subunit (the new compounds are indicated with numbers in red, while ‘n.i.’ means ‘not isolated’).

Continuing these efforts, analogues containing more electron-poor substituents were prepared to study the possible electronic effects on complexation. For this reason, the syntheses of a triple-bond- and a triazole ring-containing analogue were carried out using a *Sonogashira* and a click reaction, respectively. The synthetic routes are outlined in Scheme 2.

First, the parent acridono-crown ether (**1**) was transformed to triflate (**19**) having an excellent leaving group. This was carried out by reacting the acridono-parent compound (**1**) with triflic anhydride in the presence of *Hünig*’s base. Triflate **19** was reacted immediately without isolation in a *Sonogashira*-reaction to provide methyl ester **21** with a relatively low yield.

In the other route, the same intermediate (**19**) was subjected to a nucleophilic substitution with sodium azide to form azide **22** under phase transfer catalytic (PTC) conditions. This was required because of the poor solubility of intermediate **19** in polar solvents. It is interesting to note, that no additional phase transfer catalyst was needed since crown ether intermediate **19** could also act as a catalyst supporting the solution of the sodium azide in the organic phase. To prove this assumption, the same reaction was attempted by using the corresponding 9-triflate-activated derivative of 4,5-dimethoxyacridine, when only a negligible conversion could be reached in the same two-phase system. Azide **22** was converted without purification, as it is prone to revert to the more stable acridone form (**1**). The subsequent click reaction provided the functionalized triazole derivative (**23**) with a low yield.

The low yields (19 and 27%) are attributed to the instability of the 9-activated intermediates (**19**,**22**), which was expected from the preliminary studies on the non-cyclic analogues [25]. Esters **21** and **23** showed good stability even at room temperature in the air and can easily be transformed by a mild hydrolysis to the corresponding carboxylic acids, which are suitable for covalent immobilization.

**Figure 5:**
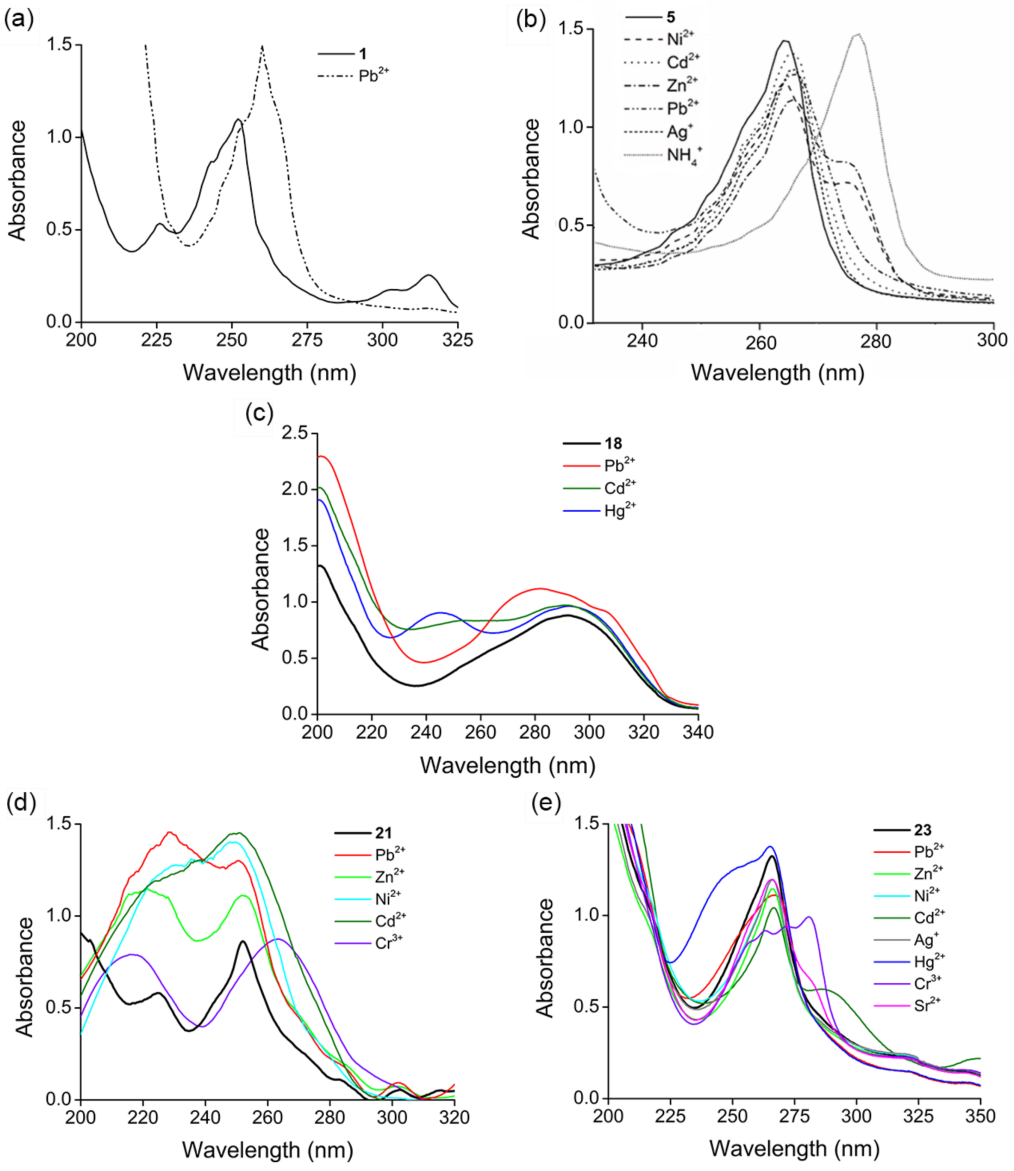
UV–Vis spectroscopy-based selectivity studies of the previously reported (**1**,**5**) and the new 9-functionalized acridino-crown ethers (**18**,**21**,**23**) toward 24 different cations [response signals of (**a**) reported acridono-parent compound (**1**, c = 100 µM) [18]; (**b**) reported 9-phenylacridino-analogue (**5**, c = 50 µM) [20]; (**c**) new crown ether **18** (c = 13 µM); (**d**) new crown ether **21** (c = 110 µM); (**e**) new crown ether **23** (c = 120 µM)]. Only those ions that induced spectral changes are indicated. The spectra of the previously reported compounds are visualized using greyscale figures.

The synthesized new crown ethers (**21**,**23**) were studied using UV–Vis spectroscopy to investigate their complexation properties toward 24 different metal ions compared to the Pb^2+^-selective parent compound (**1**). The guest cations were separately added as aqueous solutions to the acetonitrile solution of the crown ethers in a 10 molar equivalent excess in each case. If significant complexation took place, it would inevitably change the electronic structure of the host molecule, as the chromophore unit is a part of the coordination sphere. (These types of crown ethers tend to form only 1:1 complexes with cations; other complex stoichiometries and mixed binding motifs are atypical.) Consequently, the absence of changes in absorption spectra indicates the lack of molecular recognition. The results are shown in Figure 5. Only those ions that induced spectral changes are indicated in Figure 5.

It seems, that the parent compound (**1**) preferred only Pb^2+^ for complexation among the studied cations. The coordination of Pb^2+^ resulted in an increased absorbance and a slight bathochromic shift (250→265 nm) of the main excitation band, while an almost total quenching of absorbance was observed at a local excitation maximum (315 nm).

Bathochromic shifts were similarly observed upon the complexation of crown ether **5**. At the same shifted wavelength, the Ni^2+^ and Zn^2+^ complexes also showed an absorption band with a lower intensity. This crown ether (**5**) coordinated almost all of the studied cations, but most of the complexes are of relatively weak stability according to the slight spectral changes. Although the spectral changes are not in a direct correlation with the strength of the intermolecular coordination, the majority of the complexes formed with the competitive ions are not stable enough to determine the corresponding stability constants. It seemed that the selectivity of crown ether **5** is significantly decreased upon converting its acridone unit to 9-phenylacridine.

It can clearly be seen from the spectra of crown ether **18** that complexation took place with Cd^2+^ and Hg^2+^ besides the expected Pb^2+^. These competing ions have a very similar chemical character, so the obtained similar preference is not surprising. In the presence of the other cations, the initial spectrum of the free crown ether was observed. The complexation of all of the three preferred cations caused an enhancement of the absorption, especially at the lower wavelength range, without a significant shift in the spectra.

Crown ether **21** complexed Zn^2+^, Pb^2+^, Ni^2+^, Cd^2+^ and Cr^3+^ among the studied 24 metal ions. The selectivity was similarly decreased as in the case of analogue **18**. The difference is that new absorption bands appeared upon complexation.

Methyl ester-functionalized crown ether **23** also showed no selectivity. Most of the complexed cations caused no spectral shift, the shape of the initial spectrum of the free crown ether was changed only when coordinating Hg^2+^, Cr^3+^ or Cd^2+^ as the most typical competing ions.

In summary, the conversion of the acridone unit of the host molecules to 9-substituted acridines results in a reduced Pb^2+^-selectivity while inducing the coordination of many other soft electrophilic competing cations. Thus, the second approach seemed to be the better solution for modifying the oligoether unit of the macrocycles since it will not cause the loss of Pb^2+^-preference.

### 2.3. Functionalization via the Oligoether Unit (Approach 2)

It was obvious from the selectivity studies on the functionalized acridino-crown ethers that the acridone-acridine conversion of the heterocyclic unit—despite an easier synthetic route—results in the loss of Pb^2+^-selectivity. On the other hand, many similarly soft electrophilic cations were complexed due to the soft nucleophilic character of the acridine-*N* as the most important coordinative part inside the macrocycle cavity. Consequently, the heterocyclic unit should be maintained in its acridone form during the functionalization.

**Figure 6:**
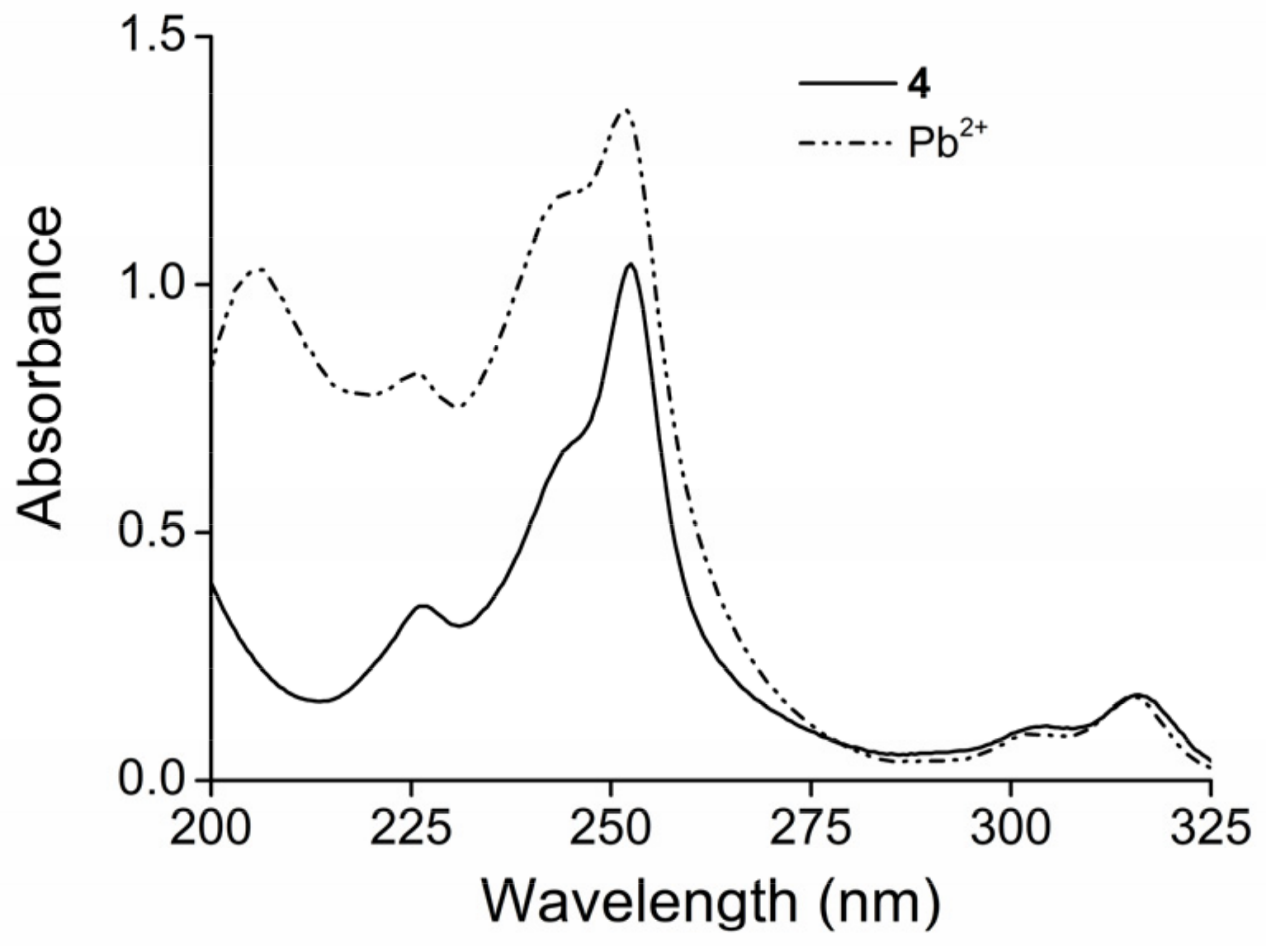
Consequent selectivity study on the previously reported lipophilic acridono-18-crown-6 ether (**4**, c = 100 µM). Only the response toward Pb^2+^ is shown, as the other 23 metal ions did not cause spectral changes.

An oligoether ring-functionalized lipophilic acridono-crown ether analogue (**4**) has already been reported recently [21]. Its selectivity was consequently restudied (Figure 6).

The study showed that this crown ether (**4**) preserved the high Pb^2+^-selectivity over the competing ions. Moreover, it was also proved that this type of structural modification did not influence the stability of the Pb^2+^-complex Thus, further efforts were focused on the suitable modification of the oligoether unit of the macrocycle. The synthesis of the oligoether intermediates required the use of orthogonal protective groups and the desired functionalization was obtained via the introduction of an *O*-benzyl unit. For providing functionalizability, the cleavage of the benzyl group can be carried out by catalytic hydrogenation, which does not change the other parts of the host molecule. It is important to note that our aim was to obtain functionalized analogues by the shortest routes using previously synthesized intermediates if possible.

**Scheme 3:**
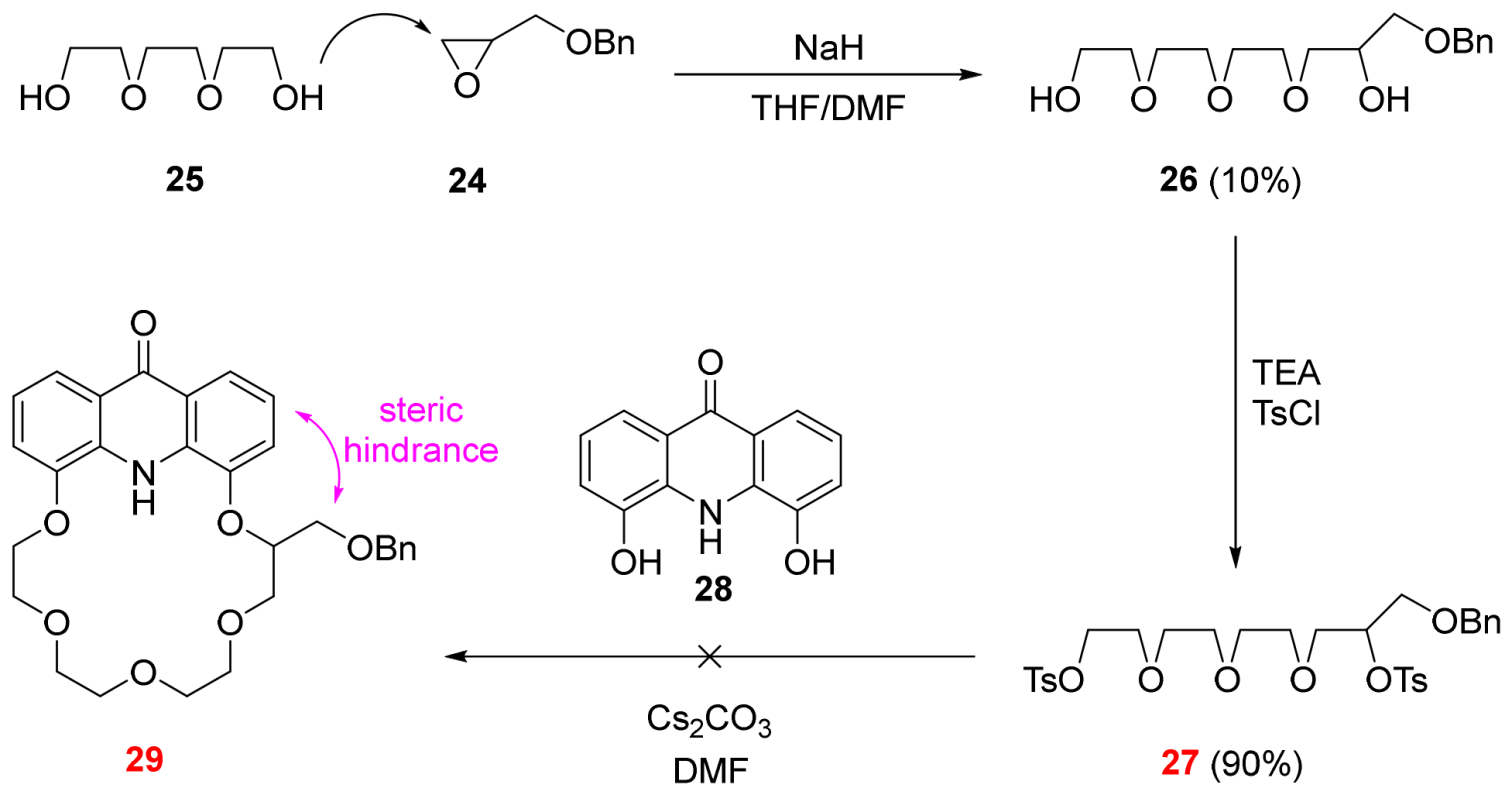
The first attempted pathway for synthesizing derivative (**29**) of the parent acridono-crown ether (**1**) functionalized at the oligoether unit (the new compounds are indicated with numbers in red).

Initially, the synthesis of a functionalizable analogue began with the opening of the benzyl glycidyl ether ring (**24**) with deprotonated triethylene glycol (**25**) based on a reported procedure [27]. The attempted synthetic route can be seen in Scheme 3.

Initially, this pathway was favored as it would result in a suitable host molecule with only three reaction steps. Moreover, the synthesis started with inexpensive and commercially available materials. However, the opening of the epoxide ring led to a low yield due to the expected regioselectivity problems and the formation of numerous byproducts. The subsequent tosylation resulted in new ditosylate **27** with a good yield. Unfortunately, the macrocyclization of ditosylates **27** with the bifunctional acridono-precursor (**28**) failed. Probably, the benzyloxymethylene substituent of tetraethylene glycol ditosylate **27** sterically hindered the ring closure.

**Scheme 4:**
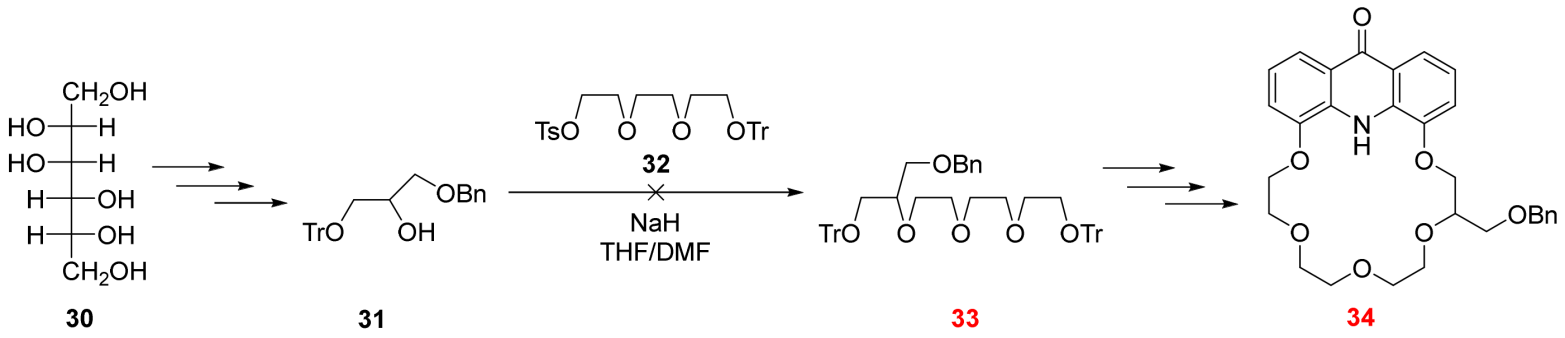
Attempted synthesis of structural analogue **34** of crown ether **29** containing the reactive linker one *C*-*C*-bond further from the rigid acridone unit (the new compounds are indicated with numbers in red).

To solve this problem, the target compound was redesigned, and the reactive linker was to be placed one *C*-*C*-bond further from the acridone unit. The revised synthetic route can be seen in Scheme 4.

**Scheme 5:**
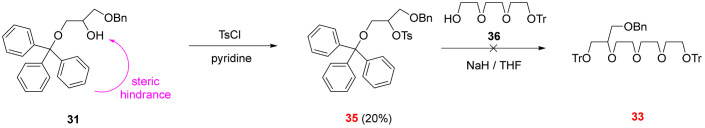
Investigating the decreased reactivity of intermediate **31** (the new compounds are indicated with numbers in red).

Reported intermediate **31** was synthesized starting from mannitol (**30**) according to a reported procedure [28,29,30]. The former was treated with monotosyl-monotrityl triethylene glycol **32** to provide ditrityl **33**, but this coupling did not succeed. Steric hindrance of the trityl group at the primary hydroxyl of **31** might have caused the failure. To prove this assumption, a test reaction was carried out for tosylating **31** (Scheme 5).

Decreased reactivity of intermediate **31** was observed even in the case of tosylation. This clearly indicates that the steric hindrance of the trityl group plays a critical role in the reactivity of the secondary hydroxyl group of intermediate **31**. It is important to consider this hindering during the design of analogues. After isolating tosylate **35** with a low yield, it was reacted further with monotritylated triethylene glycol **36**, but because of the former reason, no conversion could be reached.

**Scheme 6:**
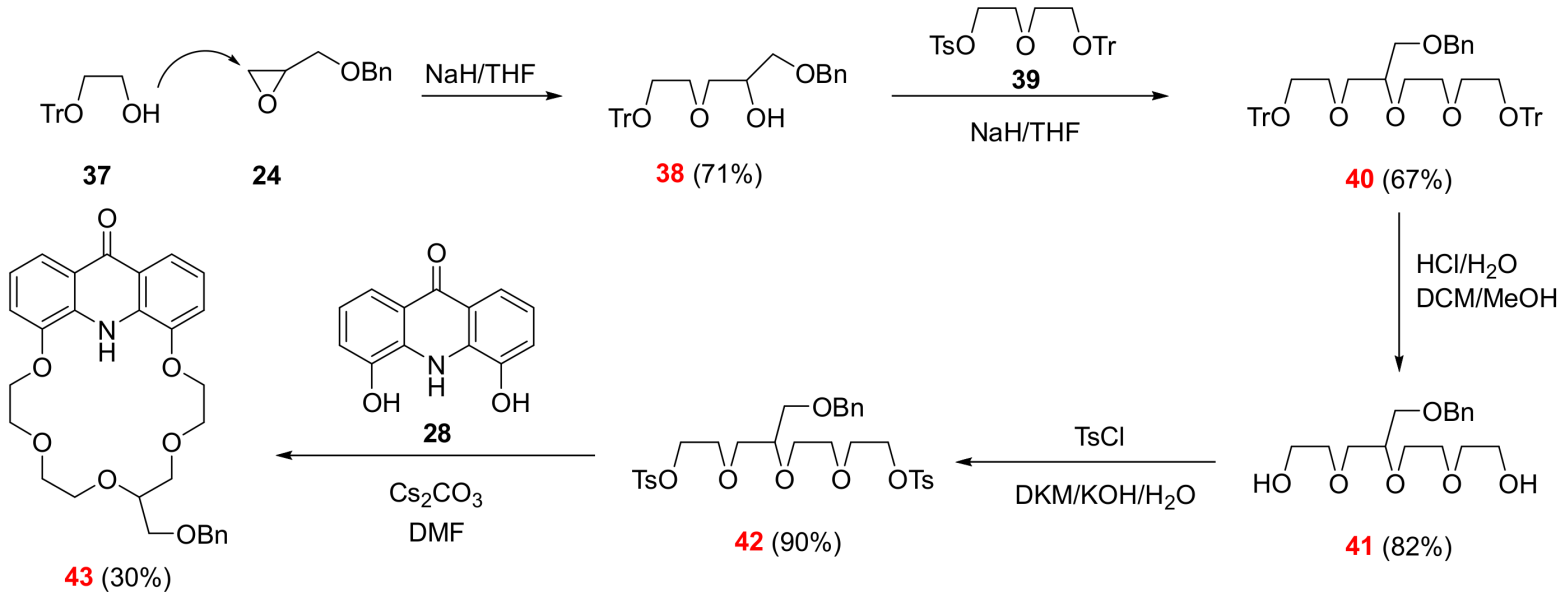
The successful synthetic pathway to obtain a crown ether analogue (**43**) with the desired functionalizability (the new compounds are indicated with numbers in red).

Further enhancing the distance between the active linker and the acridone unit helped in overcoming the previous synthetic difficulties. Finally, a longer but more reliable synthetic pathway was designed (Scheme 6).

In the first step, benzyl glycidyl ether (**24**) was reacted with monotritylated glycol **37** [31] using sodium hydride for deprotonation in tetrahydrofuran. Monohydroxy-derivative **38** was obtained with a good yield. This was then coupled under the same conditions with monotosylate **39** [32] to provide protected tetraethylene glycol **40** with a moderate yield. The trityl groups of **40** were removed by 25% aqueous hydrochloric acid in a dichloromethane methanol mixture. Tosylation of diol **41** was subsequently performed to introduce good terminal leaving groups, then key intermediate **42** was reacted with the bifunctional acridone **28**. The macrocyclization resulted in an appreciable yield when considering reported analogous reactions.

**Figure 7:**
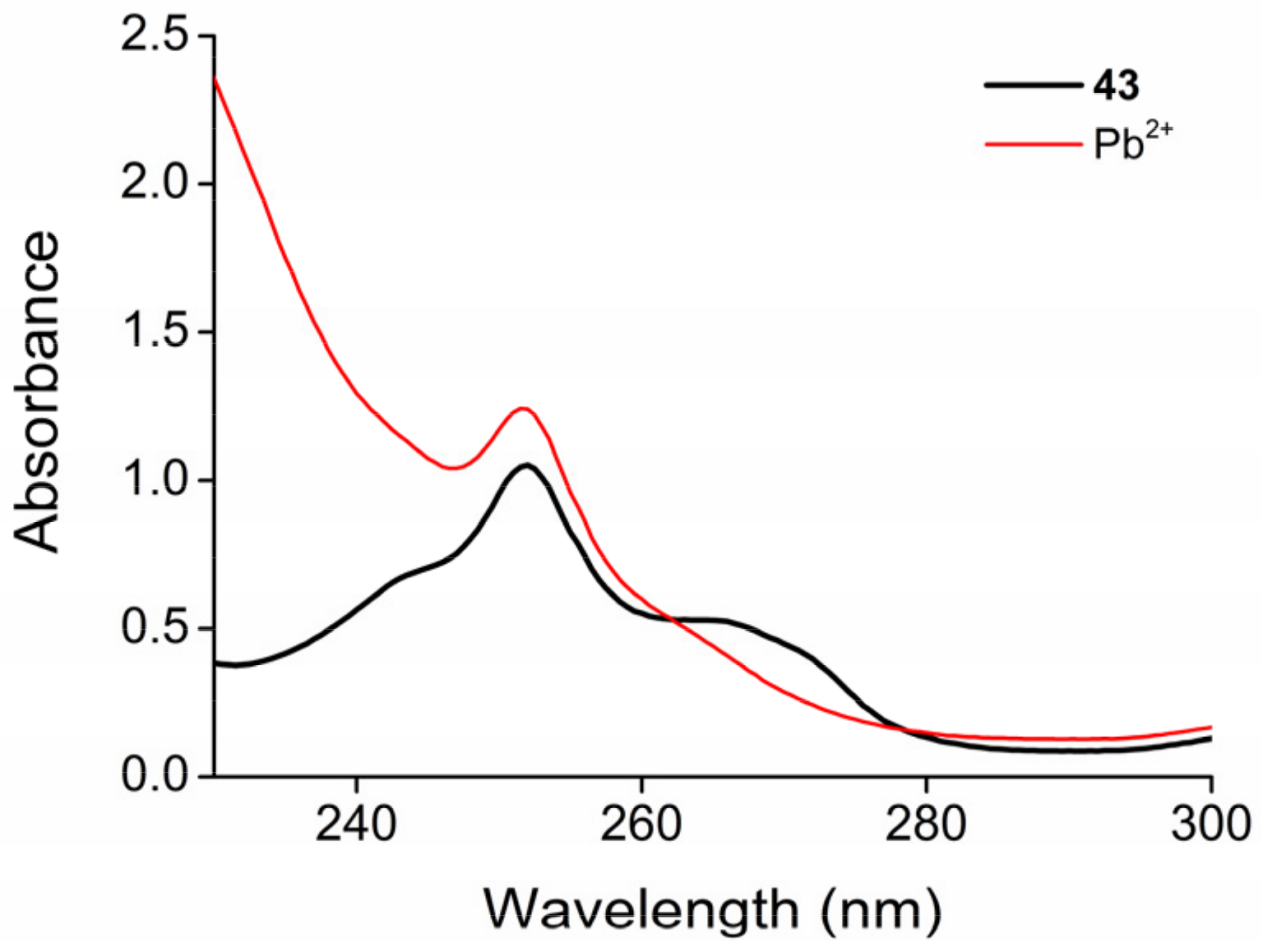
UV–Vis selectivity study of new macrocycle **43** modified at the oligoether unit toward 24 different metal ions in acetonitrile-water medium (c = 200 µM). Only the response toward Pb^2+^ was indicated, as the other 23 metal ions did not cause spectral changes.

The selectivity of the successfully prepared new macrocycle **43** was then studied and it was compared to the other functionalized analogues (Figure 7).

In the case of macrocycle **43** functionalized at the oligoether unit, the excellent selectivity of parent crown ether **1** was successfully maintained despite the structural modifications. This result supports our assumption that substituents at the oligoether unit do not alter the preference in the molecular recognition of acridono-crown ethers toward metal ions. Furthermore, complexation showed favorable reversibility, i.e., decomplexation could be performed even by a simple liquid-liquid extraction (aqueous–organic) without using any additives like EDTA.

## 3. Experimental Section

### 3.1. General

Starting materials and reagents were purchased from Sigma-Aldrich Corporation (USA, owned by Merck, Darmstadt, Germany) and used without further purification unless otherwise noted. Solvents were dried and purified according to well-established methods [33]. Silica Gel 60 F_254_ (Merck, Darmstadt, Germany) and aluminum oxide 60 F_254_ neutral type E (Merck, Germany) plates were used for thin-layer chromatography (TLC). All reactions were monitored using TLC and visualized using a UV lamp. Aluminum oxide (neutral, activated, Brockman I) and Silica Gel 60 (70–230 mesh, Merck, Darmstadt, Germany) were used for column chromatography. Ratios of solvents for the eluents are given in volumes (mL/mL). Purifications using preparative thin-layer chromatography (PTLC) were carried out using Silica gel 60 F_254_ (Merck, Germany) plates of 2 mm layer thickness (art No.: 1.05744) or aluminum oxide 60 F_254_ neutral type E (Merck, Darmstadt, Germany) plates of 0.25 mm layer thickness (art No.: 1.05727). Evaporations were carried out under reduced pressure unless otherwise stated.

The new compounds were characterized by their physical constants, such as melting point, thin-layer chromatography retention factor (*R*_f_), IR, ^1^H-NMR and ^13^C-NMR spectroscopies and HRMS spectrometry. Melting points were taken on a Boetius micro-melting point apparatus and are uncorrected. Infrared spectra were recorded on a Bruker Alpha-T FT-IR spectrometer (Bruker Corporation, Billerica, MA, USA) using KBr pastilles. ^1^H- (300 MHz) and ^13^C- (75 MHz) NMR spectra were recorded on a Bruker 300 Avance spectrometer (Bruker Corporation, Billerica, MA, USA). ^1^H- (500 MHz) and ^13^C- (125 MHz) NMR spectra were taken on a Bruker DRX-500 Avance spectrometer (Bruker Corporation, Billerica, MA, USA). HRMS analysis was carried out on a Thermo Velos Pro Orbitrap Elite (Thermo Fisher Scientific, Dreieich, Germany) system. The ionization method was ESI and was operated in positive ion mode. The protonated molecular ion peak was fragmented using CID at a normalized collision energy of 35–45%. The sample was dissolved in methanol. Data acquisition and analysis were accomplished with Xcalibur software version 2.2 (Thermo Fisher Scientific, Dreieich, Germany).

UV–Vis spectra were recorded on a UNICAM UV4-100 spectrophotometer controlled using VIZION 3.4 software (ATI UNICAM, Hatley Saint George, UK). Quartz cuvettes with a path length of 1 cm were used in all cases. Spectroscopic measurements were carried out at room temperature (25 ± 1 °C). During the selectivity studies, the aqueous solutions of the metal salts (10 equivalent) were added with a *Hamilton*-syringe to the acetonitrile solutions of the macrocycles. The initial concentration of the free host compounds was adjusted to obtain an absorption maximum of 1.0–1.5 for better visual comparability. The reported spectra were corrected in each case with the background signal of the added solutions and concentration values were also corrected corresponding to the caused dilution. OriginPro 8.6 (OriginLab Corp., Northampton, MA, USA) software was used for the evaluation and visualization of the spectroscopic results.

### 3.2. Synthesis of the New Compounds

#### 3.2.1. 27-[4-(Diethoxymethyl)phenyl]-6,9,12,15,18-pentaoxa-25-azatetracyclo[21.3.1.0^5^,^26^.0^19^,^24^]heptacosa-1,3,5(26),19,21,23(27),24-heptaene (See **16** in Scheme 1)

Acridono-18-crown-6 ether **1** (100 mg, 0.26 mmol) was converted into its chloro-acridino-derivative **14** according to a reported procedure [25]. *Grignard*-reagent **15** was prepared from 1-bromo-4-(diethoxymethyl)benzene (672 mg, 2.60 mmol) [26].

A solution of crude chloroacridine **14** (105 mg, 0.26 mmol) in a mixture of dry and pure toluene (15 mL) was added dropwise to a stirred solution of *Grignard*-reagent **15** (2.60 mmol in 10 mL THF), palladium acetate (4 mg, 0.018 mmol), dilithium tetrachlorocuprate (1.0 M in THF, 6 µL, 0.026 mmol) under an argon atmosphere at room temperature. The temperature of the resulting reaction mixture was raised to 60 °C and kept at this temperature for 20 h then cooled down to 20 °C. The solvent was removed, and the residue was taken up in ethyl acetate (25 mL) and ice-cold water (25 mL). The pH of the aqueous phase was adjusted to 7 using aqueous hydrochloric acid (5 m/m%). The phases were shaken well and separated. The aqueous phase was extracted with ethyl acetate (3 × 15 mL). The combined organic phase was dried over magnesium sulfate, filtered and the solvent was removed. The crude product was purified using PTLC on neutral aluminum-oxide adsorbent using an eluent mixture of ethanol:toluene 1:50 to provide the title compound **16** as a yellow solid (13 mg, 9%).

M.p. = 113 °C. *R*_f_ = 0.49 (Al_2_O_3_, ethanol:toluene 1:20). ^1^H-NMR (CDCl_3_): δ [ppm]: 7.83 (d, *J* = 8.2 Hz, 2H, ArCH), 7.78 (d, *J* = 8.2 Hz, 2H, ArCH), 7.66 (d, *J* = 8.2 Hz, 2H, ArCH), 7.62 (d, *J* = 8.2 Hz, 2H, ArCH), 7.38 (t, *J* = 8.2 Hz, 2H, acridine ArCH), 5.59 (s, 1H, protected formyl CH), 3.76–3.52 (m, 20H, ethereal OCH_2_), 1.29 (t, *J* = 7.1 Hz, 6H, acetal CH_3_). ^13^C-NMR (CDCl_3_): δ [ppm]: 158.47, 139.27, 135.99, 135.24, 130.36, 130.28, 128.03, 127.68, 127.36, 127.21, 126.63, 101.24, 64.90, 61.19, 61.03, 58.43, 15.24. IR: ν_max_ [cm^−1^]: 3350 (broad, in the case of the hemiacetal **17**), 3064, 1563, 1512, 1449, 1390, 1313, 1281, 1210, 1169, 1106, 1090, 1007, 975, 861, 813, 736, 659, 604, 551, 500, 408. HRMS: m/z = [MH^+^]: 548.2572, (Calculated for C_32_H_37_NO_7_, 547.2570).

#### 3.2.2. 4-{6,9,12,15,18-Pentaoxa-25-azatetracyclo[21.3.1.0^5^,^26^.0^19^,^24^]heptacosa-1,3,5(26),19,21,23(27),24-heptaen-27-yl}benzaldehyde (See **18** in Scheme 1)

Aqueous hydrochloric acid solution (1 mL, 10 m/m%) was added dropwise to a stirred solution of the diacetal-protected crown ether **16** (13 mg, 0.024 mmol) in acetone (500 µL). The reaction mixture was stirred at 50 °C for 3 h under argon. The solvent was evaporated to provide the title compound **18** as a yellow crystal (12 mg, quantitative yield).

M.p. = 144 °C. *R*_f_ = 0.47 (Al_2_O_3_, ethanol:toluene 1:20). ^1^H-NMR (CDCl_3_): δ [ppm]: 10.07 (s, 1H, formyl H), 7.98 (dd, *J* = 8.7, 4.6 Hz, 2H, ArCH), 7.94 (dd, *J* = 8.9, 4.5 Hz, 2H, ArCH), 7.79 (dd, *J* = 8.9, 4.5 Hz, 2H, ArCH), 7.74 (dd, *J* = 8.2, 4.7 Hz, 2H, ArCH), 7.34 (t, *J* = 8.0 Hz, 2H, acridine ArCH), 3.71–3.49 (m, 16H, ethereal OCH_2_). ^13^C-NMR (CDCl_3_): δ [ppm]: 190.71, 157.28, 138.08, 134.80, 134.05, 129.17, 129.08, 126.84, 126.48, 126.16, 126.02, 125.44, 63.71, 60.00, 59.83. IR: ν_max_ [cm^−1^]: 2964, 2924, 2841, 2742, 1934, 1691, 1602, 1562, 1514, 1449, 1390, 1313, 1282, 1211, 1169, 1106, 1090, 1007, 975, 861, 813, 736, 659, 604, 551, 500. HRMS: *m*/*z* = [MH^+^]: 474.1836, (Calculated for C_28_H_27_NO_6_, 473.1838).

#### 3.2.3. 6,9,12,15,18-Pentaoxa-25-azatetracyclo[21.3.1.0^5^,^26^.0^19^,^24^]heptacosa-1,3,5(26),19,21,23(27),24-heptaen-27-yl trifluoromethanesulfonate (See **19** in Scheme 2)

Acridono-crown ether (**1**, 50 mg, 0.13 mmol) was dissolved in dry dichloromethane (10 mL) in a flame-dried flask equipped with a septum and an argon inlet. The solution was cooled to 0 °C by using an external ice bath. Then, triflic anhydride (66 µL, 110 mg, 0.39 mmol) was added to the stirred solution using a syringe. *Hünig*’s base (136 µL, 101 mg, 0.78 mmol) was also added using a syringe and the reaction mixture was stirred for 30 min. The volatile components were removed at 20 °C. (If the reaction mixture was poured onto a large excess of 40 m/m% aqueous tetramethylammonium hydroxide at 0 °C and was extracted with dichloromethane, the majority of the product was hydrolyzed). Based on TLC analysis, a quantitative conversion was achieved and the crude product as an orange solid was further reacted without purification to gain functionalized crown ether **21**.

#### 3.2.4. Methyl 4-(2-{6,9,12,15,18-pentaoxa-25-azatetracyclo[21.3.1.0^5^,^26^.0^19^,^24^]heptacosa-1,3,5(26),19,21,23(27),24-heptaen-27-yl}ethynyl)benzoate (See **21** in Scheme 2)

Freshly prepared triflate (**19**) (60 mg, 0.12 mmol) was suspended in dry acetonitrile (2.0 mL). Copper(I) bromide (10 mg, 0.07 mmol), tetrakis(triphenylphosphine)palladium(0) (12 mg, 0.01 mmol) and diisopropylamine (842 µL, 6.00 mmol) were added to the suspension, which was stirred at room temperature for 15 min. Acetylene **20** (100 mg, 0.62 mmol) dissolved in acetonitrile (2.0 mL) was added to the stirred reaction mixture at 0 °C under argon. The temperature of the reaction mixture was raised to room temperature and stirred at this temperature for 48 h under argon. The volatile components were removed. The residue was dissolved in a mixture of ethyl acetate (10 mL) and water (10 mL). The phases were shaken well and separated. The aqueous phase was extracted with ethyl acetate (5 × 10 mL). The combined organic phase was dried over magnesium sulfate, filtered and the solvent was removed. The crude product was purified using column chromatography on aluminum oxide adsorbent using a gradient elution of toluene and ethanol (0–10% ethanol). The product needed further purification using PTLC on silica gel using dichloromethane as an eluent to provide **21** (12 mg, 19%) as a yellow solid.

M.p. = 98–99 °C. *R*_f_ = 0.30 (Al_2_O_3_, ethanol:toluene 1:20). *R*_f_ = 0.20 (SiO_2_, dichloromethane). *R*_f_ = 0.87 (SiO_2_, methanol:dichloromethane 1:10). ^1^H-NMR (CDCl_3_): δ [ppm]: 8.09–7.83 (m, 2H, ArCH), 7.63–7.54 (m, 2H, ArCH), 7.50–7.40 (m, 2H, ArCH), 7.35–7.26 (m, 2H, ArCH), 7.19–7.09 (m, 2H, ArCH), 4.47–4.29 (m, 3H, ethereal OCH_2_), 4.07–3.77 (m, 12H, ethereal OCH_2_ and ester OCH_3_), 3.61–3.34 (m, 4H, ethereal OCH_2_). ^13^C-NMR (CDCl_3_): δ [ppm]: 166.23, 146.54, 132.45, 131.44, 130.56, 129.56, 126.09, 120.72, 118.84, 113.13, 81.86, 76.27, 71.37, 69.44, 52.34. IR: ν_max_ [cm^−1^]: 3420, 3057, 2962, 2927, 2873, 2850, 1719, 1624, 1598, 1577, 1533, 1437, 1262, 1222, 1153, 1100, 1029, 802, 748, 722, 694, 638, 573, 541, 517. HRMS: *m*/*z* = [MH^+^]: 528.1948, (Calculated for C_31_H_29_NO_7_, 527.1944).

#### 3.2.5. 27-Azido-6,9,12,15,18-pentaoxa-25-azatetracyclo[21.3.1.0^5^,^26^.0^19^,^24^]heptacosa-1,3,5(26),19,21,23(27),24-heptaene (See **22** in Scheme 2)

Triflate **19** was prepared according to the procedure described in Section 3.2.3 starting from acridono-crown ether **1** (50 mg, 0.13 mmol).

Freshly prepared triflate (**19**) (60 mg, 0.12 mmol) was suspended in a mixture of dichloromethane (2.0 mL), water (2.0 mL) and *Hünig*’s base (63 µL, 0.36 mmol). Sodium azide (78 mg, 1.2 mmol) was added to this suspension at room temperature, then the reaction mixture was stirred at 40 °C for 4 h. The mixture was cooled to room temperature, diluted with water (15 mL) and extracted with dichloromethane (3 × 5.0 mL). The combined organic phase was dried over magnesium sulfate, filtered and the solvent was removed. Based on TLC analysis a quantitative conversion was achieved and the crude product as an orange solid was further reacted without purification to gain functionalized crown ether **23**.

#### 3.2.6. Methyl 4-(1-{6,9,12,15,18-pentaoxa-25-azatetracyclo[21.3.1.0^5^,^26^.0^19^,^24^]heptacosa-1,3,5(26),19,21,23(27),24-heptaen-27-yl}-1H-1,2,3-triazol-4-yl)benzoate (See **23** in Scheme 2)

Freshly prepared crude azide (**22**) (50 mg, 0.12 mmol) was suspended in dry acetonitrile (3.0 mL). Copper(I) bromide (10 mg, 0.07 mmol) and acetylene **20** (100 mg, 0.62 mmol) were added to the suspension, which was stirred at room temperature for 15 min under argon. The temperature of the reaction mixture was raised to 50 °C and it was stirred at this temperature for 24 h under argon. The solvent was removed. The residue was dissolved in a mixture of ethyl acetate (10 mL) and water (10 mL). The phases were shaken well and separated. The aqueous phase was extracted with ethyl acetate (5 × 10 mL). The combined organic phase was dried over magnesium sulfate, filtered and the solvent was removed. The crude product was purified using column chromatography on aluminum oxide adsorbent using a gradient elution of toluene and ethanol mixture (0–10% ethanol). The product was further purified using silica gel PTLC using dichloromethane as an eluent to provide **23** (18 mg, 27%) as a yellow solid.

M.p. = 117–118 °C. *R*_f_ = 0.27 (Al_2_O_3_, ethanol:toluene 1:20). *R*_f_ = 0.16 (SiO_2_, dichloromethane). *R*_f_ = 0.78 (SiO_2_, methanol:dichloromethane 1:10). ^1^H-NMR (CDCl_3_): δ [ppm]: 8.37 (s, 1H, triazole ArCH), 8.19 (d, *J* = 8.0 Hz, 2H, ArCH), 8.11 (d, *J* = 8.1 Hz, 2H, Ar CH), 7.58–7.41 (m, 2H, ArCH), 7.12 (d, *J* = 7.1 Hz, 2H, ArCH), 7.05 (d, *J* = 8.8 Hz, 2H, ArCH), 4.74–4.68 (m, 4H, ethereal OCH_2_), 4.62–4.56 (m, 4H, ethereal OCH_2_), 3.96 (s, 3H, ester OCH_3_), 3.94–3.89 (m, 4H, ethereal OCH_2_), 3.83–3.76 (m, 4H, ethereal OCH_2_). ^13^C-NMR (CDCl_3_): δ [ppm]: 166.71, 141.10, 140.59, 132.95, 130.39, 129.76, 128.53, 127.05, 65.23, 58.41, 52.35. IR: ν_max_ [cm^−1^]: 3359 (broad, complexed water), 3112, 3040, 3018, 2962, 2929, 2872, 1718, 1628, 1604, 1483, 1449, 1261, 1156, 1098, 1078, 1016, 864, 800, 750, 745, 681, 638, 551, 459, 442, 405. HRMS: *m*/*z* = [MH^+^]: 571.2110, (Calculated for C_31_H_30_N_4_O_7_, 570.2114).

#### 3.2.7. 4,14-Bis[(4-methylbenzenesulfonyl)oxy]-1-phenyl-2,6,9,12-tetraoxatetradecane (See **27** in Scheme 3)

Diol **26** (1.00 g, 3.18 mmol) was dissolved in triethylamine (10 mL) and a solution of tosyl chloride (1.52 g, 7.95 mmol) in triethylamine (10 mL) was added dropwise to its stirred solution. The reaction mixture was stirred at room temperature for 2 days. The volatile components were removed and the residue was dissolved in a mixture of ethyl acetate (100 mL) and water (200 mL). The phases were shaken well and separated. The pH of the aqueous phase was adjusted to 7 with aqueous hydrochloric acid (5 m/m%). The aqueous phase was extracted with ethyl acetate (3 × 100 mL). The combined organic phase was extracted with saturated aqueous sodium chloride solution (100 mL), dried over magnesium sulfate, filtered and the solvent was removed. The crude product was purified using column chromatography on silica gel using ethyl acetate:hexane 1:5 mixture as eluent to provide **27** (1.78 g, 90%) as a colorless oil.

*R*_f_ = 0.42 (SiO_2_, ethyl acetate:hexane 1:4). ^1^H-NMR (CDCl_3_): δ [ppm]: 7.71 (d, *J* = 8.1 Hz, 4H, Ts ArCH), 7.32–7.08 (m, 9H, Ts and Bn ArCH), 4.65 (q, *J* = 5.1 Hz, 1H, ethereal OCH), 4.34 (d, *J* = 3.7 Hz, 2H, ethereal OCH_2_), 4.17–4.09 (m, 4H, ethereal OCH_2_), 3.63–3.58 (m, 6H, ethereal OCH_2_), 3.56–3.52 (m, 3H, ethereal OCH_2_), 3.49–3.45 (m, 3H, ethereal OCH_2_), 2.32 (s, 6H, Ts *p*-CH_3_). ^13^C-NMR (CDCl_3_): δ [ppm]: 176.33, 171.13, 144.54, 137.69, 134.00, 129.60, 129.57, 128.35, 128.00, 127.71, 127.61, 125.31, 79.65, 73.32, 70.97, 70.60, 70.57, 70.44, 69.78, 69.14, 68.67, 63.63, 21.65, 20.97. IR: ν_max_ [cm^−1^]: 2871, 1735, 1598, 1453, 1362, 1236, 1175, 1097, 1048, 921, 815, 776, 665, 553. HRMS: *m*/*z* = [MH^+^]: 623.1980, (Calculated for C_30_H_38_O_10_S_2_, 622.1906).

#### 3.2.8. General Procedure for Macrocyclizations

A mixture of dihydroxy derivative (1.00 eq.), tetraethylene glycol ditosylate (1.00 eq.) and finely powdered anhydrous caesium carbonate (8.00 eq.) in dry DMF (200 mL/g diol) was stirred vigorously under argon atmosphere at room temperature for 7 days. Water (200 mL/g diol) was added to the reaction mixture, and it was extracted with ethyl acetate (3 × 200 mL/g diol). The combined organic phase was extracted with aqueous lithium bromide solution (6 × 200 mL/g diol, 5 m/m%) and then with saturated aqueous sodium chloride solution (2 × 200 mL/g diol). The organic phase was dried over magnesium sulfate, filtered and the solvent was evaporated.

#### 3.2.9. 1-(Benzyloxy)-3-(triphenylmethoxy)propan-2-yl 4-methylbenzene-1-sulfonate (See **35** in Scheme 5)

Alcohol **31** (500 mg, 1.18 mmol) was dissolved in dry pyridine (10 mL) and a solution of tosyl chloride (677 mg, 3.54 mmol) in dry pyridine (10 mL) was added dropwise to its stirred solution. The reaction mixture was stirred at 40 °C for 3 days. The solvent was removed and the residue was dissolved in a mixture of ethyl acetate (100 mL) and water (200 mL). The phases were shaken well and separated. The pH of the cold aqueous phase was adjusted to 7 with cold aqueous hydrochloric acid (5 m/m%). The aqueous phase was extracted with ethyl acetate (3 × 100 mL). The combined organic phase was extracted with saturated aqueous sodium chloride solution (100 mL), dried over magnesium sulfate, filtered and the solvent was removed. The crude product was purified using PTLC on silica gel using ethyl acetate:hexane 1:4 mixture as eluent to provide **35** (136 mg, 20%) as a colorless oil.

*R*_f_ = 0.29 (SiO_2_, ethyl acetate:hexane 1:4). ^1^H-NMR (CDCl_3_): δ [ppm]: 7.71 (dd, *J* = 18.7, 7.6 Hz, 4H, Ts ArCH), 7.29–7.24 (m, 6H, Tr ArCH), 7.23–7.11 (m, 12H, Tr and Bn ArCH), 7.11–7.01 (m, 2H, ArCH), 4.70–4.55 (m, 1H, ethereal OCH), 4.38–4.29 (m, 1H, ethereal OCH_2_), 4.10–3.99 (m, 3H, ethereal OCH_2_), 3.62–3.54 (m, 1H, ethereal OCH_2_), 3.28–3.20 (m, 1H, ethereal OCH_2_), 2.38 (s, 3H, Ts *p*-CH_3_). ^13^C-NMR (CDCl_3_): δ [ppm]: 146.88, 143.48, 137.68, 133.36, 129.82, 129.63, 128.63, 128.29, 127.93, 127.87, 127.82, 127.08, 86.86, 80.04, 73.25, 68.85, 66.79, 21.64. IR: ν_max_ [cm^−1^]: 1598, 1494, 1448, 1355, 1189, 1176, 1096, 1003, 919, 816, 764, 702, 663, 555. HRMS: *m*/*z* = [MH^+^]: 579.2130, (Calculated for C_36_H_34_O_5_S, 578.2127).

#### 3.2.10. ({2-[3-(Benzyloxy)-2-hydroxypropoxy]ethoxy}diphenylmethyl)benzene (See **38** in Scheme 6)

Sodium hydride (1.58 g, 39.5 mmol, 60 m/m% dispersion in mineral oil) was suspended in dry tetrahydrofuran (30 mL) under argon at room temperature. Solution of alcohol **37** (10 g, 32.9 mmol, [31]) in tetrahydrofuran (50 mL) was added dropwise to the stirred suspension of sodium hydride. The resulting mixture was refluxed for 30 min. The temperature of the reaction mixture was set to 0 °C with an external ice-water bath, then a solution of benzyl-glycidyl ether **24** (5.4 g, 32.9 mmol) in tetrahydrofuran (50 mL) was added dropwise and it was stirred for 1 h at this temperature. The temperature of the reaction mixture was raised to boiling point and it was refluxed for 24 h. The solvent was evaporated, and the residue was dissolved in a mixture of cold water (100 mL) and diethyl ether (100 mL). The aqueous phase was extracted with diethyl ether (2 × 100 mL). The combined organic phase was dried over magnesium sulfate, filtered and evaporated. The crude product was purified using column chromatography on silica gel using a mixture of ethyl acetate:hexane 1:4 to provide the title compound **38** (10.9 g, 71%) as a colorless oil.

*R*_f_ = 0.20 (SiO_2_, ethyl acetate:hexane 1:4). ^1^H-NMR (CDCl_3_): δ [ppm]: 7.47 (d, *J* = 7.5 Hz, 6H, Tr ArCH), 7.39–7.18 (m, 14H, Tr and Bn ArCH), 4.57 (s, 2H, Bn OCH_2_C), 4.03 (t, *J* = 5.4 Hz, 1H, ethereal OCH_2_), 3.81–3.45 (m, 6H, ethereal OCH_2_ and OCH), 3.25 (t, *J* = 4.9 Hz, 2H, ethereal OCH_2_), 2.61 (s, 1H, OH). ^13^C-NMR (CDCl_3_): δ [ppm]: 147.00, 144.11, 128.76, 128.51, 128.02, 127.95, 127.86, 127.26, 73.52, 72.79, 72.50, 71.33, 69.68, 61.68. IR: ν_max_ [cm^−1^]: 3447 (broad), 3085, 3058, 3030, 2918, 2868, 1596, 1490, 1448, 1217, 1076, 1002, 950, 900, 761, 745, 695, 649, 632, 465. HRMS: *m*/*z* = [MH^+^]: 469.2353, (Calculated for C_31_H_32_O_4_, 468.2301).

##### 3.2.11. 7-[(Benzyloxy)methyl]-1,1,1,15,15,15-hexaphenyl-2,5,8,11,14-pentaoxapentadecane (See **40** in Scheme 6)

Sodium hydride (390 mg, 16.3 mmol, 60 m/m% dispersion in mineral oil) was suspended in dry tetrahydrofuran (7 mL) under argon at room temperature. Solution of alcohol **38** (5.45 g, 10.8 mmol) in tetrahydrofuran (30 mL) was added dropwise to the stirred suspension of sodium hydride. The resulting mixture was refluxed for 30 min. The temperature of the reaction mixture was set to 0 °C with an external ice-water bath, then a solution of tosylate **39** (5.08 g, 10.8 mmol, [32]) in tetrahydrofuran (30 mL) was added dropwise and it was stirred for 1 h at this temperature. The temperature of the reaction mixture was raised to 40 °C and it was stirred for 5 days. The solvent was evaporated, and the residue was mixed with ice-cold water (100 mL), then this aqueous mixture was extracted with ethyl acetate (3 × 100 mL). The combined organic phase was dried over magnesium sulfate, filtered and evaporated. The crude product was purified using column chromatography on silica gel adsorbent using a gradient elution of ethyl acetate and hexane (0–20% ethyl acetate) to provide the title compound **40** (5.78 g, 67%) as a colorless oil.

*R*_f_ = 0.40 (SiO_2_, ethyl acetate:hexane 1:4). ^1^H-NMR (CDCl_3_): δ [ppm]: 7.43–7.35 (m, 12H, Tr ArCH), 7.23–7.15 (m, 23H, Tr and Bn ArCH), 4.51–4.42 (m, 2H, ethereal OCH_2_), 3.73–3.50 (m, 13H, ethereal OCH_2_ and OCH), 3.21–3.11 (m, 4H, ethereal OCH_2_). ^13^C-NMR (CDCl_3_): δ [ppm]: 146.95, 144.18, 138.03, 129.73, 128.78, 128.48, 127.99, 127.96, 127.80, 127.71, 127.28, 126.98, 86.58, 82.05, 78.48, 73.52, 73.05, 72.93, 69.76, 69.71, 63.40, 61.60, 61.50. IR: ν_max_ [cm^−1^]: 3087, 3060, 3031, 2916, 2870, 2246, 1597, 1490, 1446, 1328, 1078, 1031, 1002, 906, 727, 696, 648, 633, 583, 509, 448. HRMS: *m*/*z* = [MH^+^]: 799.3921, (Calculated for C_54_H_54_O_6_, 798.3920).

###### 3.2.12. 2-[3-(Benzyloxy)-2-[2-(2-hydroxyethoxy)ethoxy]propoxy]ethan-1-ol (See **41** in Scheme 6)

Aqueous hydrochloric acid solution (20 mL, 25 m/m%) was added slowly to a stirred mixture of protected tetraethylene glycol **40** (2.50 g, 3.19 mmol) in dichloromethane (20 mL) and methanol (20 mL). The reaction mixture was stirred at room temperature for 1 day under argon. The volatile components were evaporated, and the residue was taken up in a mixture of ice-cold water (100 mL) and diethyl ether (100 mL). The phases were shaken well and separated. The aqueous phase was extracted with diethyl ether (5 × 100 mL). The combined organic phase was dried over magnesium sulfate, filtered and evaporated to provide title compound **41** (820 mg, 82%) as a colorless oil.

*R*_f,**40**_ = 0.90 (SiO_2_, ethyl acetate). *R*_f,**41**_ = 0.33 (SiO_2_, ethyl acetate, phosphomolybdic acid stain). ^1^H-NMR (CDCl_3_): δ [ppm]: 7.46–7.30 (m, 1H, Bn ArCH), 7.29–7.16 (m, 4H, Bn ArCH), 4.50 (s, 2H, Bn OCH_2_C), 4.18–4.14 (m, 2H, ethereal OCH_2_), 3.67–3.53 (m, 15H, ethereal OCH_2_ and OCH), 2.76 (broad s, 2H, OH). ^13^C-NMR (CDCl_3_): δ [ppm]: 138.12, 128.35, 127.64, 127.62, 78.44, 73.39, 72.69, 71.34, 71.12, 70.76, 70.65, 69.77, 69.31, 69.07, 63.58, 61.61. IR: ν_max_ [cm^−1^]: 3468 (broad), 2868 (broad), 1735, 1453, 1370, 1233, 1101, 1052, 958, 885, 739, 699, 606. HRMS: *m*/*z* = [MH^+^]: 315.1728, (Calculated for C_16_H_26_O_6_, 314.1729).

###### 3.2.13. 1-({2-[3-(Benzyloxy)-2-(2-{2-[(4-methylbenzenesulfonyl)oxy]ethoxy}ethoxy)propoxy]ethoxy}sulfonyl)-4-methylbenzene (See **42** in Scheme 6)

Diol **41** (480 mg, 1.53 mmol) was dissolved in dichloromethane (10 mL) under argon. The temperature of this solution was set to 0 °C with an external ice-water bath, then a cold aqueous solution of potassium hydroxide (20 mL, 40 m/m%) was added to it. The resulting mixture was stirred at 0 °C for 10 min. A solution of tosyl chloride (730 mg, 3.82 mmol) in dichloromethane (10 mL) was added dropwise, then the mixture was stirred at room temperature for 5 days. Water (50 mL) and dichloromethane (50 mL) were added to the reaction mixture, the pH of the aqueous phase was adjusted to 8 with aqueous hydrochloric acid (5 m/m%), then the phases were shaken well and separated. The aqueous phase was further extracted with dichloromethane (3 × 50 mL). The combined organic phase was dried over magnesium sulfate, filtered and evaporated. The crude product was purified using column chromatography on silica gel adsorbent using a gradient elution of ethyl acetate and hexane (0–20% ethyl acetate) to provide the title compound **42** (857 mg, 90%) as a colorless oil.

*R*_f_ = 0.41 (SiO_2_, ethyl acetate:hexane 1:1). ^1^H-NMR (CDCl_3_): δ [ppm]: 7.82–7.79 (m, 4H, Ts ArCH), 7.38–7.31 (m, 9H, ArCH), 4.53 (s, 2H, Bn OCH_2_C), 4.16–4.11 (m, 4H, ethereal OCH_2_), 3.71–3.66 (m, 6H, ethereal OCH_2_), 3.63–3.54 (m, 5H, ethereal OCH_2_ and OCH), 3.53–3.50 (m, 2H, ethereal OCH_2_), 2.45 (s, 6H, tosyl *p*-CH_3_). ^13^C-NMR (CDCl_3_): δ [ppm]: 144.78, 138.22, 133.06, 128.37, 127.96, 127.63, 78.42, 73.39, 71.46, 70.93, 69.86, 69.84, 69.29, 69.20, 68.88, 68.65, 21.62. IR: ν_max_ [cm^−1^]: 2871, 1735, 1598, 1453, 1362, 1236, 1175, 1097, 1048, 921, 815, 776, 665, 606, 553. HRMS: m/z = [MH^+^]: 623.1908, (Calculated for C_30_H_38_O_10_S_2_, 622.1906).

###### 3.2.14. 13-[(Benzyloxy)methyl]-6,9,12,15,18-pentaoxa-25-azatetracyclo[21.3.1.0^5^,^26^.0^19^,^24^]heptacosa-1,3,5(26),19,21,23-hexaen-27-one (See **43** in Scheme 6)

Acridono-crown ether **43** was prepared according to the general procedure described in Section 3.2.8. starting from the previously reported acridone diol **28** (1.00 g, 4.42 mmol, [18]) and ditosylate **42** (2.75 g, 4.42 mmol). The crude product was purified using column chromatography on silica gel using a gradient elution of dichloromethane and methanol (0–5% methanol). The product needed further purification using PTLC on neutral aluminum oxide using an eluent mixture of ethanol:toluene 1:50. Finally, it was recrystallized from isopropyl alcohol to provide the title compound **43** (670 mg, 30%) as a yellow solid.

M.p. = 63 °C. *R*_f_ = 0.41 (SiO_2_, methanol:dichloromethane 1:30). *R*_f_ = 0.53 (Al_2_O_3_, ethanol:toluene 1:20). ^1^H-NMR (CDCl_3_): δ [ppm]: 9.39 (s, 1H, acridone NH), 8.07 (d, *J* = 8.2 Hz, 2H, acridone CH), 7.32–7.30 (m, 5H, Bn CH), 7.17 (t, *J* = 8.0 Hz, 2H, acridone CH), 7.10–7.06 (m, 2H, acridone CH), 4.54 (s, 2H, Bn OCH_2_C), 4.39–4.29 (m, 5H, ethereal OCH_2_), 4.12–3.98 (m, 3H, ethereal OCH_2_), 3.94–3.90 (m, 2H, ethereal OCH_2_), 3.84–3.77 (m, 4H, ethereal OCH_2_), 3.71–3.52 (m, 3H, ethereal OCH_2_ and OCH). ^13^C-NMR (CDCl_3_): δ [ppm]: 177.93, 162.51, 146.71, 138.15, 131.39, 128.34, 127.59, 122.11, 120.66, 118.65, 112.30, 112.08, 78.52, 73.42, 72.90, 71.80, 69.97, 69.92, 69.81, 69.38, 68.84. IR: ν_max_ [cm^−1^]: 2961, 2923, 2653, 2739, 1694, 1602, 1561, 1493, 1465, 1389, 1312, 1259, 1207, 1167, 1087, 1014, 797, 659, 550, 476. HRMS: *m*/*z* = [MH^+^]: 506.2107, (Calculated for C_29_H_31_NO_7_, 505.2101).

## 4. Conclusions

We have reported herein different synthetic approaches for modifying the structural backbone of a Pb^2+^-selective acridono-crown ether with outstanding properties to provide its covalent immobilization on various supports. Moreover, an extended study was carried out on the structure–applicability relationships within this group of host molecules, including a comparison with all of the relevant, previously reported analogues.

**Figure 8:**
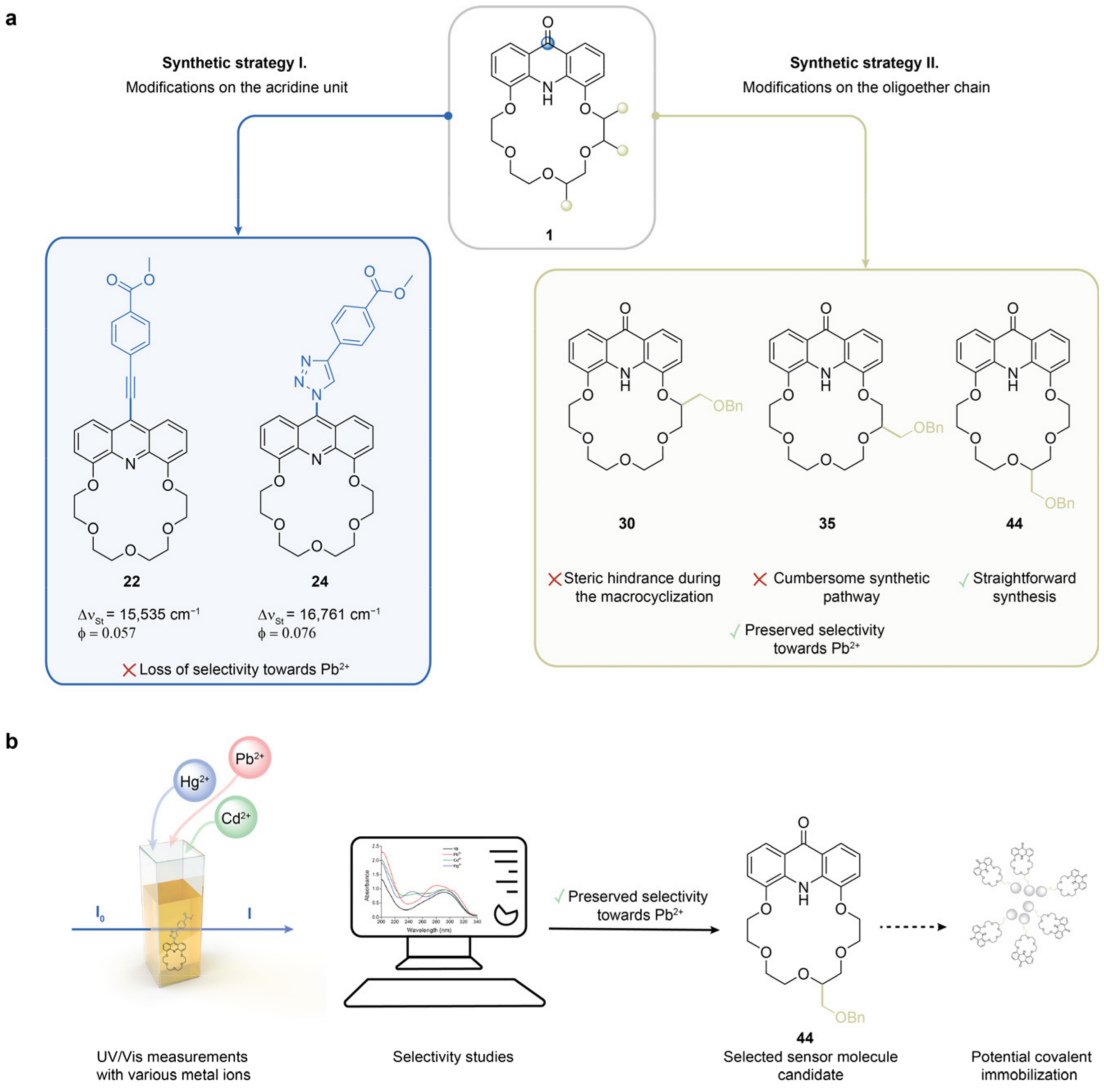
The main outcomes and conclusions of the present study (**a**) Synthetic strategies; (**b**) Optochemical sensing.

In summary, oligoether-unit-modified analogues are preferred to preserve the excellent Pb^2+^-selectivity of the parent acridono-crown ether. If the functionalization was carried out at position *9* of the heterocyclic unit, the selectivity is expected to be decreased. (In the case of modifying the acridone unit at other positions, the expected regioselectivity is quite low and typically harsher reaction conditions are needed). These non-selective analogues also preferred some other soft nucleophilic heavy metal cations (like Hg^2+^, Cr^3+^ Cd^2+^, etc.). In contrast, the modification of the oligoether unit did not influence the molecular recognition properties of the parent compound. The present extended study supports the generalizability of these statements. Finally, an oligoether-modified methylene benzyloxy derivative was obtained, which showed high Pb^2+^-selectivity, reversibility (possible decomplexation by extraction with water) and stability. Figure 8 summarizes the main outcomes and conclusions of the work.

Recent supramolecular applications have clearly demonstrated the prominent place of the proposed type of crown ethers among alternative host molecules. We believe that the presented results, considerations and suggestions will contribute to the future design and development of sensor and selector molecules with outstanding applicability.

## Data Availability

The original contributions presented in the study are included in the article/Supplementary Materials, further inquiries can be directed to the corresponding author/s.

## Supplementary Materials

The following supporting information can be downloaded at: https://www.mdpi.com/article/10.3390/molecules29051121/s1, Figure S1: ^1^H-NMR spectrum for **16** (CDCl_3_); Figure S2: ^13^C-NMR spectrum for **16** (CDCl_3_); Figure S3: ^1^H-NMR spectrum for **18** (CDCl_3_); Figure S4: ^13^C-NMR spectrum for **18** (CDCl_3_); Figure S5: ^1^H-NMR spectrum for **21** (CDCl_3_); Figure S6: ^13^C-NMR spectrum for **21** (CDCl_3_); Figure S7: ^1^H-NMR spectrum for **23** (CDCl_3_); Figure S8: ^13^C-NMR spectrum for **23** (CDCl_3_); Figure S9: ^1^H-NMR spectrum for **27** (CDCl_3_); Figure S10: ^13^C-NMR spectrum for **27** (CDCl_3_); Figure S11: ^1^H-NMR spectrum for **35** (CDCl_3_); Figure S12: ^13^C-NMR spectrum for **35** (CDCl_3_); Figure S13: ^1^H-NMR spectrum for **38** (CDCl_3_); Figure S14: ^13^C-NMR spectrum for **38** (CDCl_3_); Figure S15: ^1^H-NMR spectrum for **40** (CDCl_3_); Figure S16: ^13^C-NMR spectrum for **40** (CDCl_3_); Figure S17: ^1^H-NMR spectrum for **41** (CDCl_3_ + D_2_O); Figure S18: ^13^C-NMR spectrum for **41** (CDCl_3_); Figure S19: ^1^H-NMR spectrum for **42** (CDCl_3_); Figure S20: ^13^C-NMR spectrum for **42** (CDCl_3_); Figure S21: ^1^H-NMR spectrum for **43** (CDCl_3_); Figure S22: ^13^C-NMR spectrum for **43** (CDCl_3_).
