# Supplementary material for: Synthetic Modifications of a Pb2+-Sensor Acridono-Crown Ether for Covalent Attachment and Their Effects on Selectivity
Source: Molecules. 2024 Mar 1;29(5):1121. doi: 10.3390/molecules29051121 (PMC10935152; doi:10.3390/molecules29051121)
Supplement: Supplementary file 1 [file molecules-29-01121-s001.zip › molecules-2877316-supplementary.pdf]

Supplementary Material

Synthetic Modifications of a Pb^{2+}-Sensor Acridono-Crown Ether for Covalent Attachment and Their Effects on Selectivity

Panna Vezse [1], Martin Gede [1,2], Ádám Golcs [1,3,*], Péter Huszthy [1] and Tünde Tóth [1,4,*]

[1] Department of Organic Chemistry and Technology, Budapest University of Technology and Economics, Szent Gellért Sq. 4, H-1111 Budapest, Hungary
[2] Advanced Membranes and Porous Materials Center, Physical Science and Engineering Division, King Abdullah University of Science and Technology, Thuwal 23955-6900, Saudi Arabia
[3] Department of Pharmaceutical Chemistry, Semmelweis University, Hőgyes Endre Str. 9, H-1092 Budapest, Hungary
[4] HUN-REN Centre for Energy Research, Konkoly-Thege Miklós Str. 29-33, H-1121 Budapest, Hungary
* Correspondence: golcs.adam@semmelweis.hu (Á.G.); toth.tunde@vbk.bme.hu (T.T.)

Contents

1. ^{1}H- and ^{13}C-NMR spectra of the new compounds
2. Investigated ions in selectivity studies

1. ^{1}H- and ^{13}C-NMR spectra of the new compounds

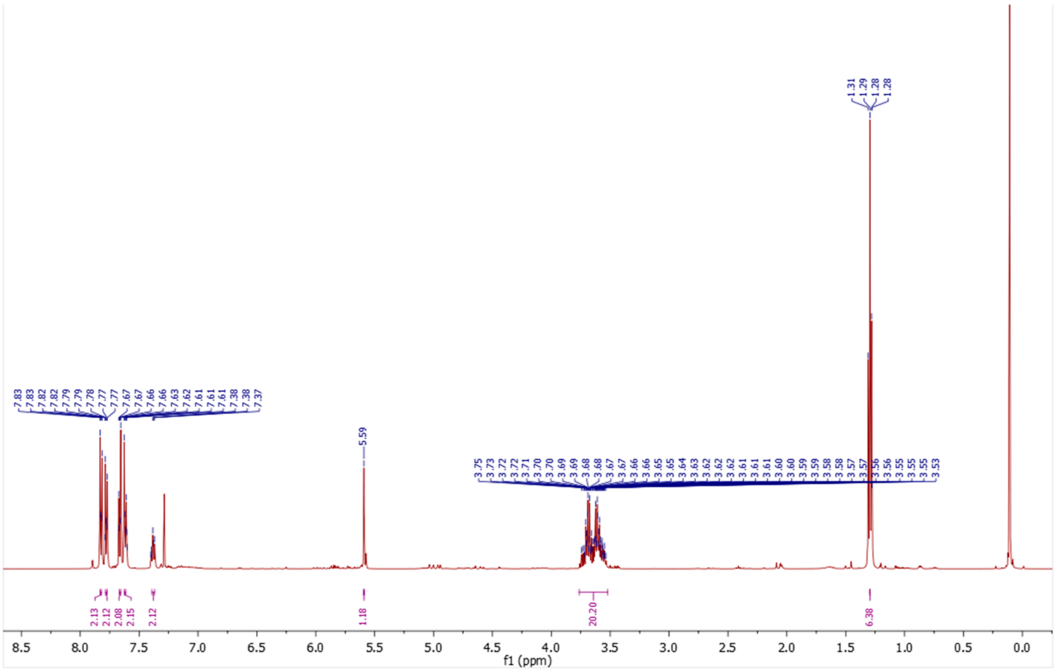

Figure S1. ^{1}H-NMR spectrum for **16** ($CDCl_3$)

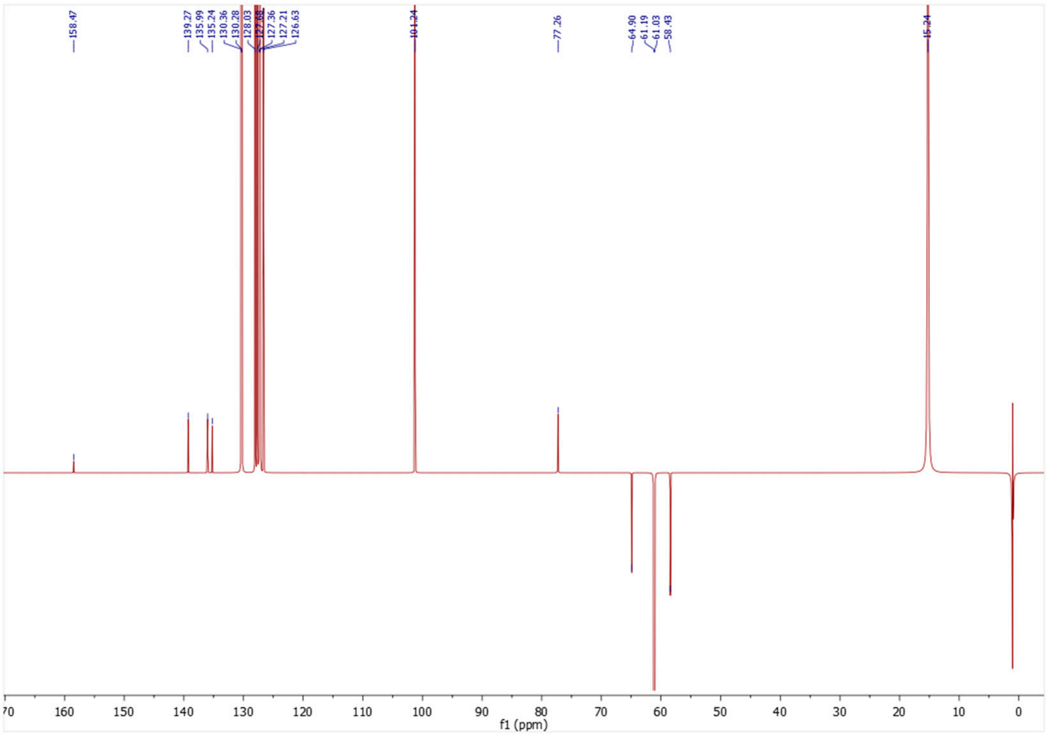

Figure S2. ^{13}C-NMR spectrum for **16** ($CDCl_3$)

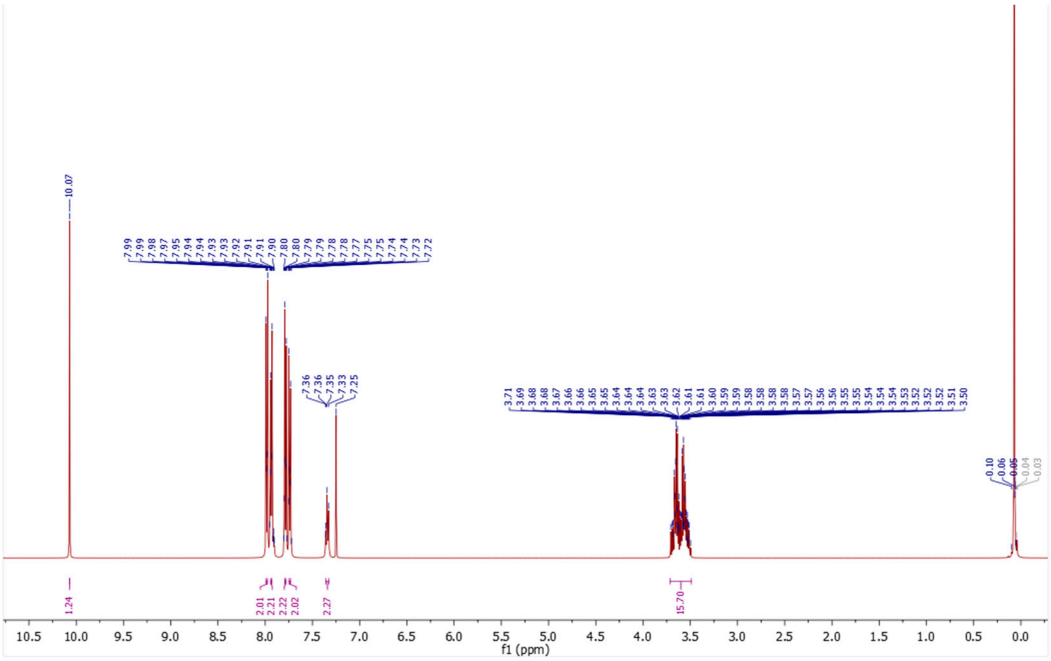

Figure S3. ^{1}H-NMR spectrum for **18** ($CDCl_3$)

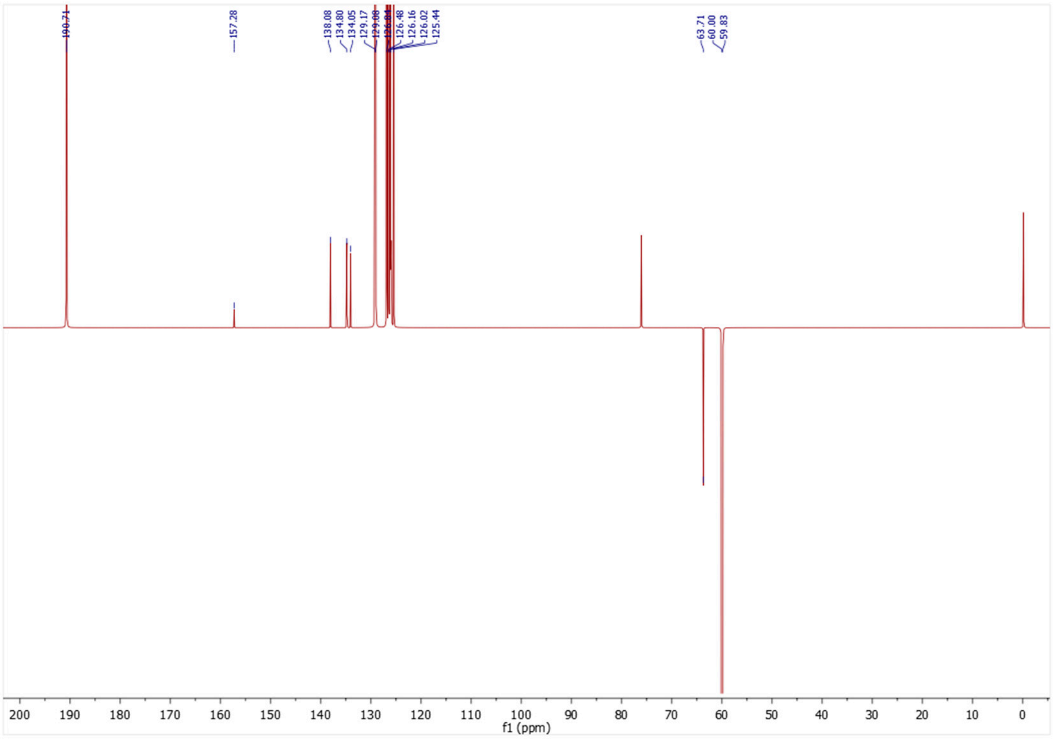

Figure S4. ^{13}C-NMR spectrum for **18** ($CDCl_3$)

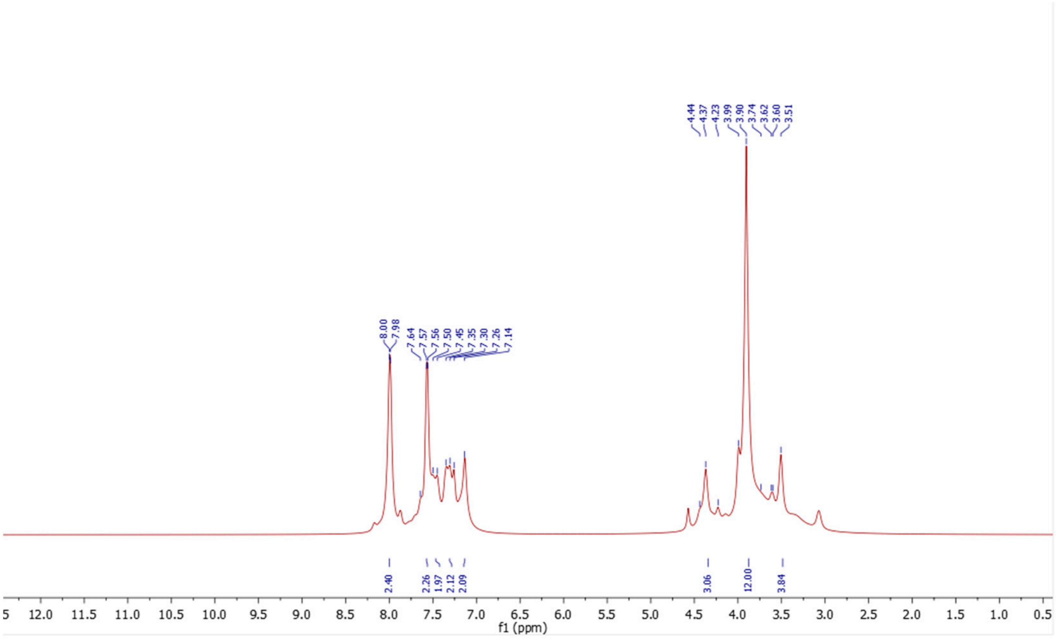

Figure S5. ^{1}H-NMR spectrum for **21** ($CDCl_3$)

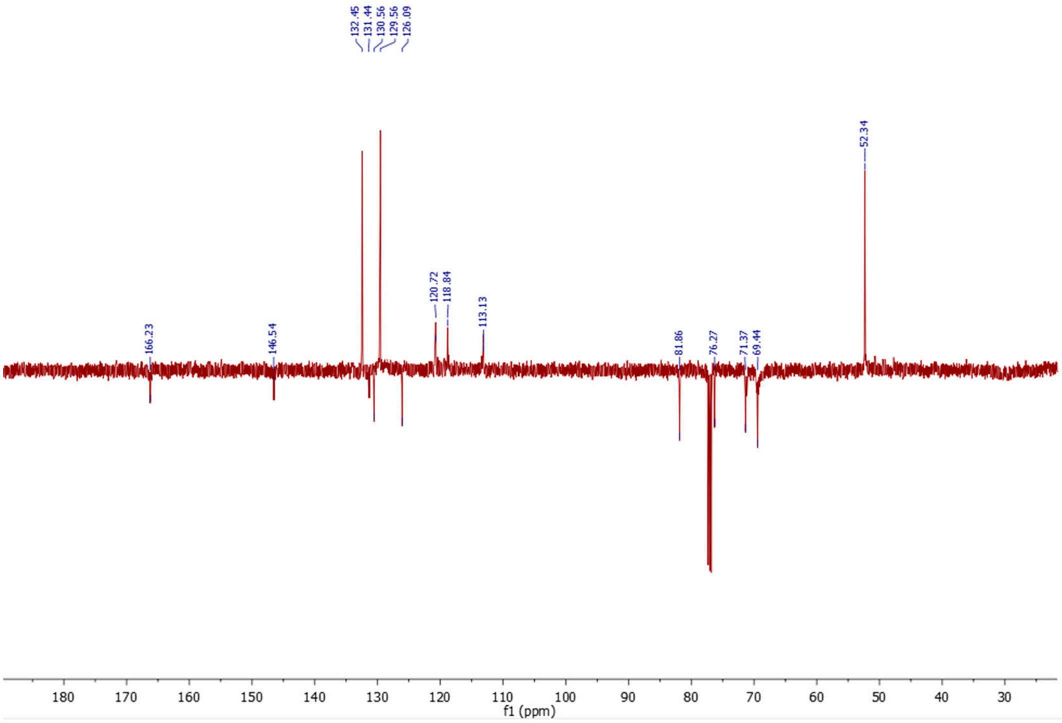

Figure S6. ^{13}C-NMR spectrum for **21** ($CDCl_3$)

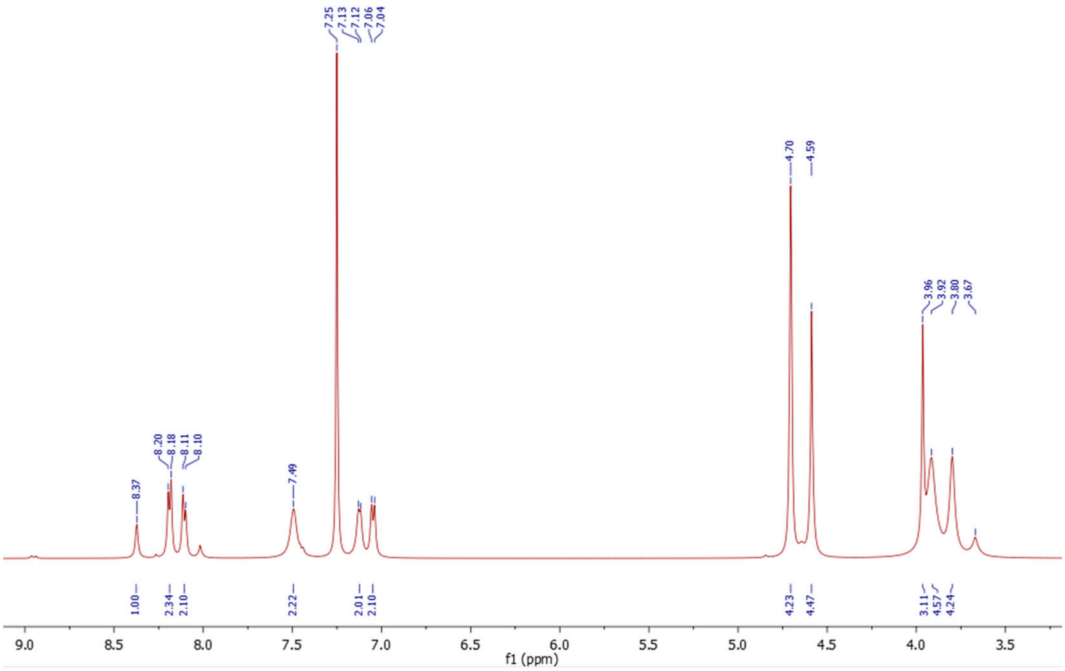

Figure S7. ^{1}H-NMR spectrum for **23** ($CDCl_3$)

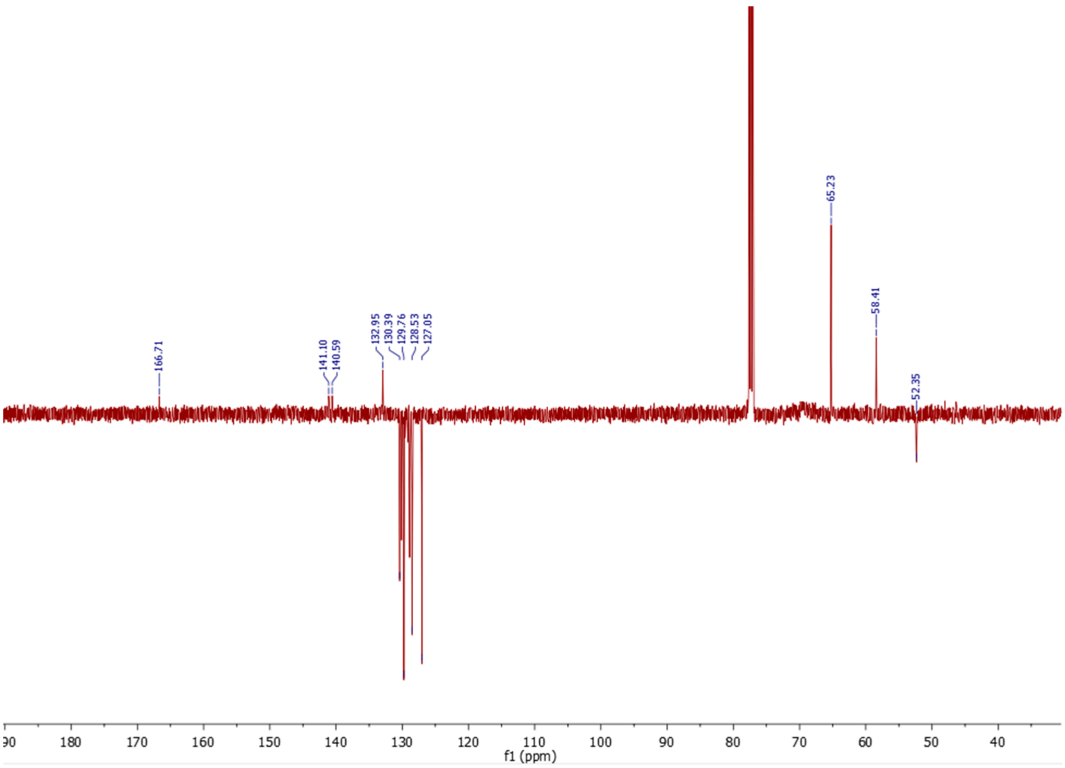

Figure S8. ^{13}C-NMR spectrum for **23** ($CDCl_3$)

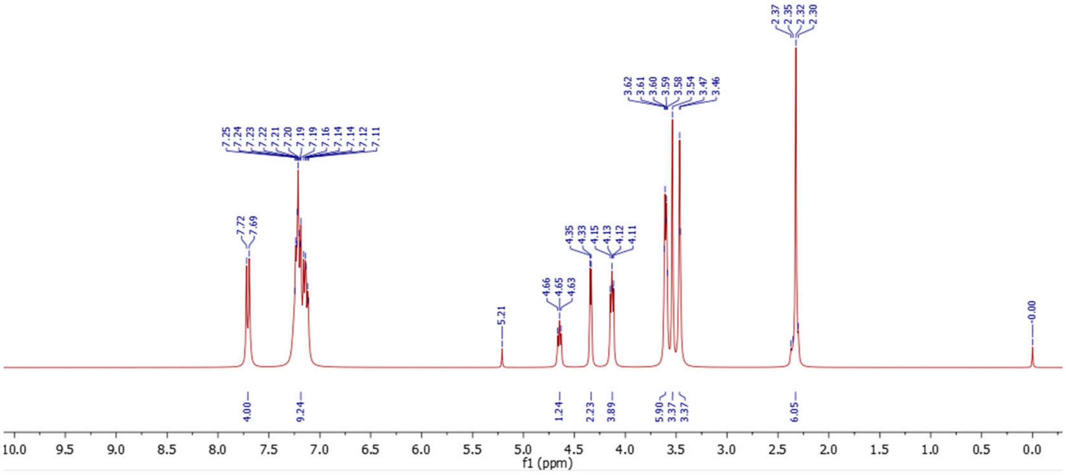

Figure S9. ^{1}H-NMR spectrum for **27** ($CDCl_3$)

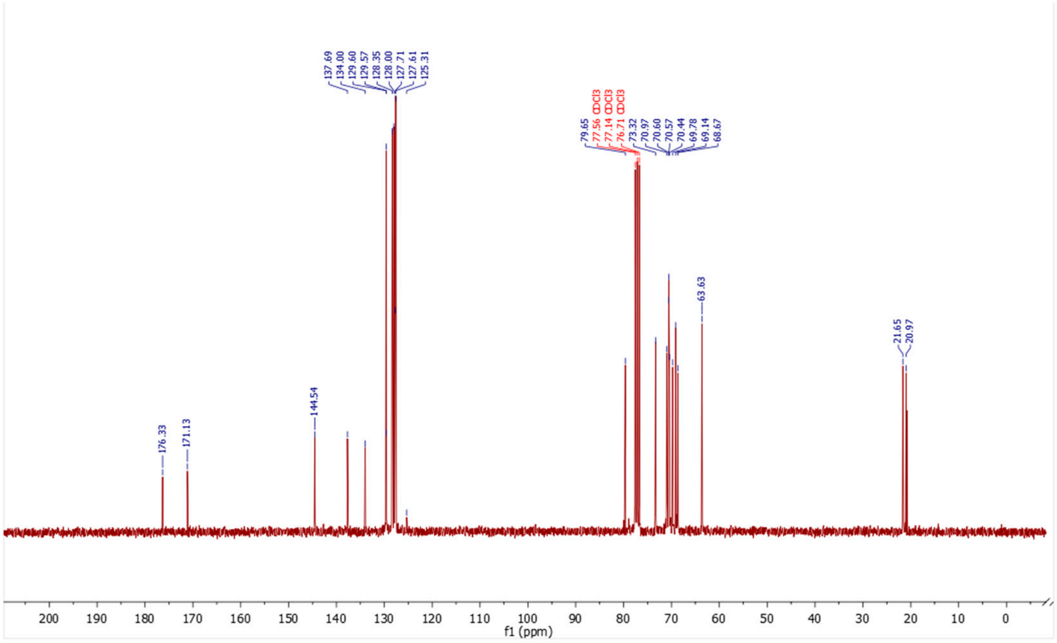

Figure S10. ^{13}C-NMR spectrum for **27** ($CDCl_3$)

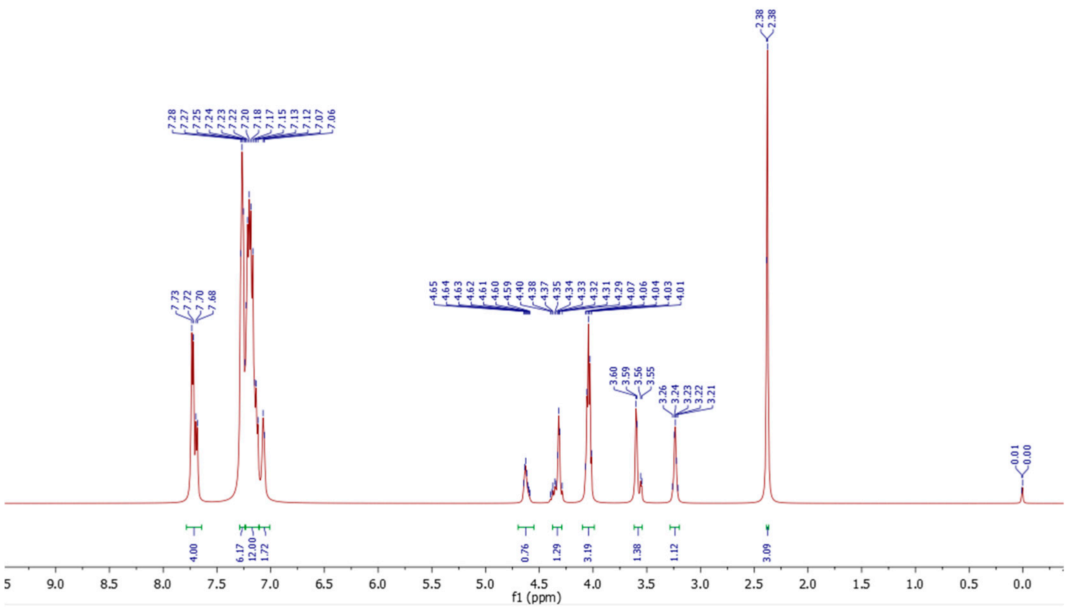

Figure S11. ^{1}H-NMR spectrum for **35** ($CDCl_3$)

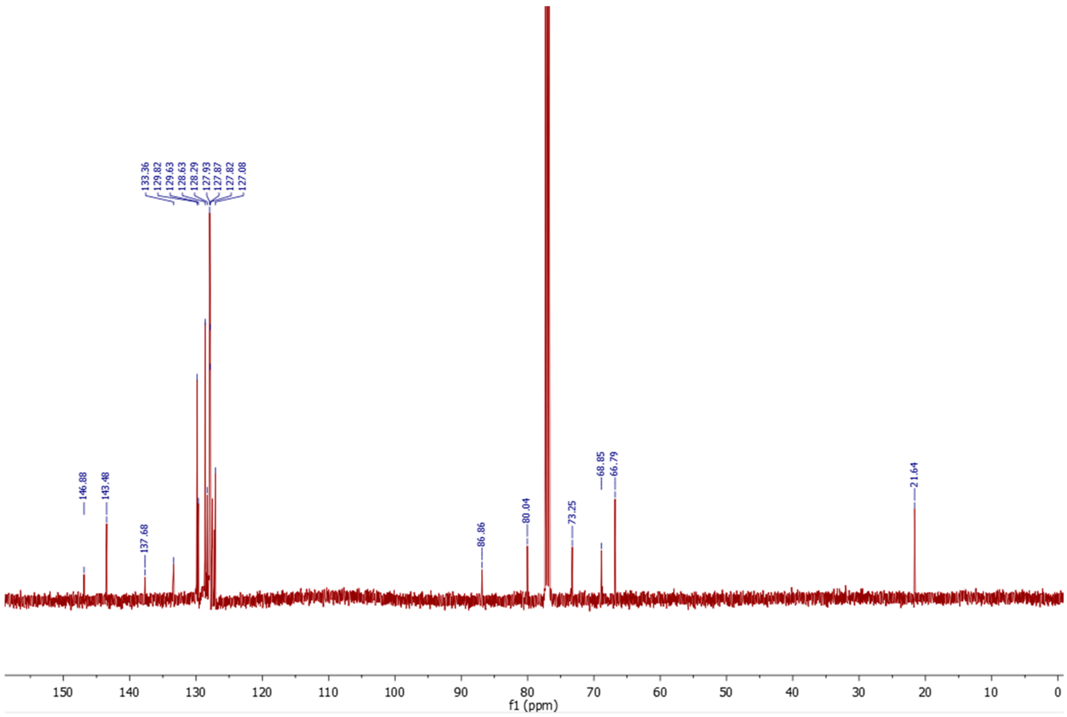

Figure S12. ^{13}C-NMR spectrum for **35** ($CDCl_3$)

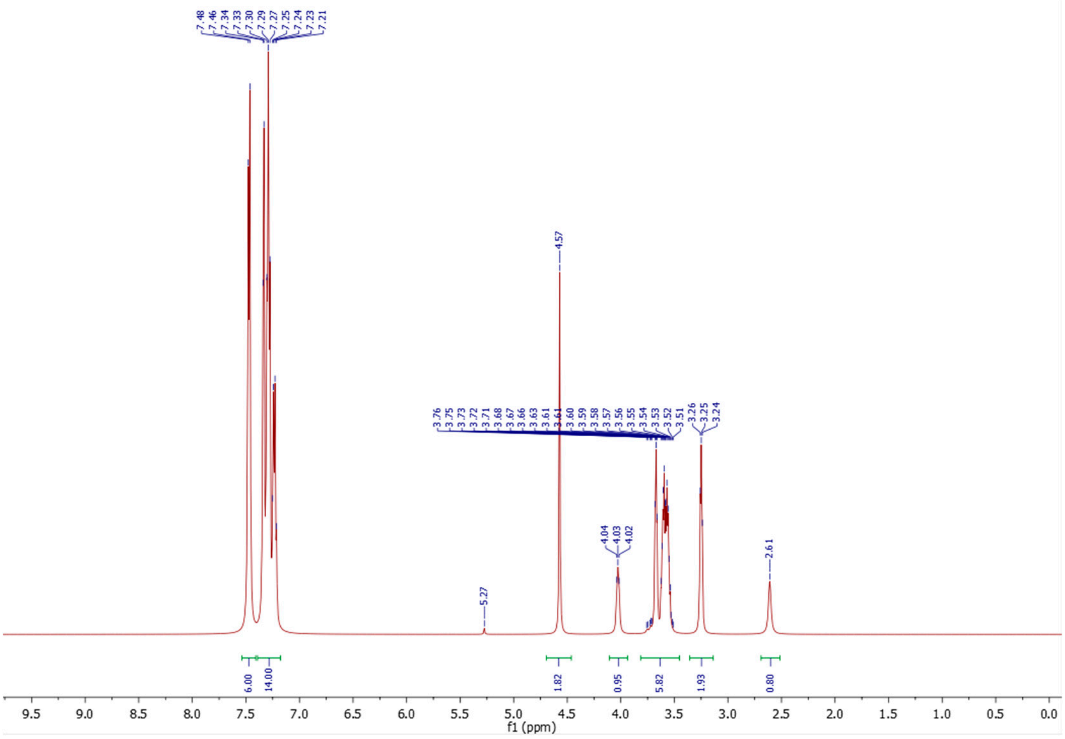

Figure S13. ^{1}H-NMR spectrum for **38** ($CDCl_3$)

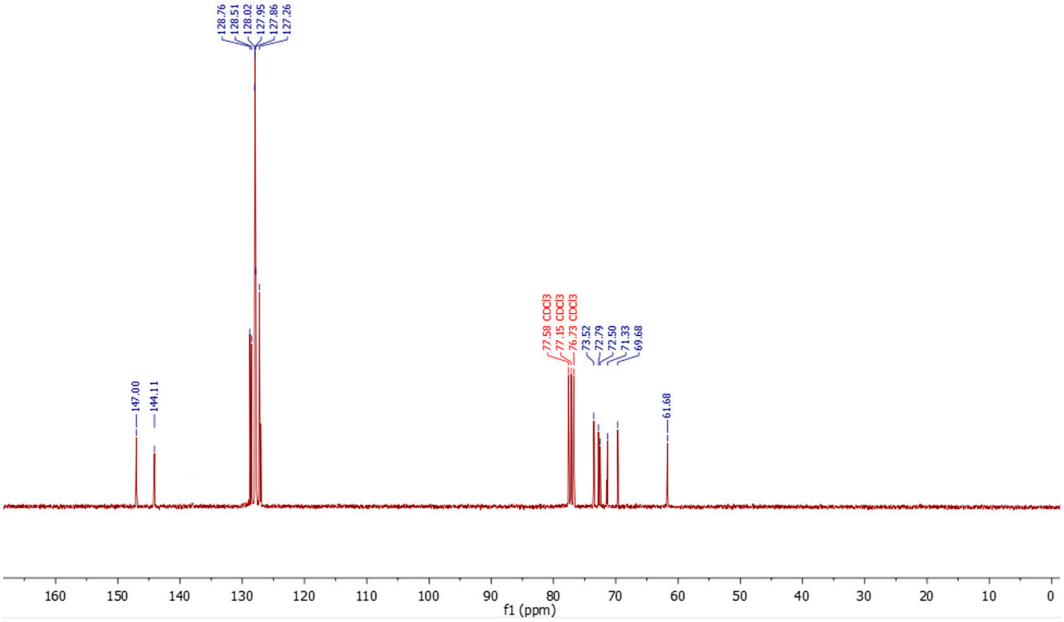

Figure S14. ^{13}C-NMR spectrum for **38** ($CDCl_3$)

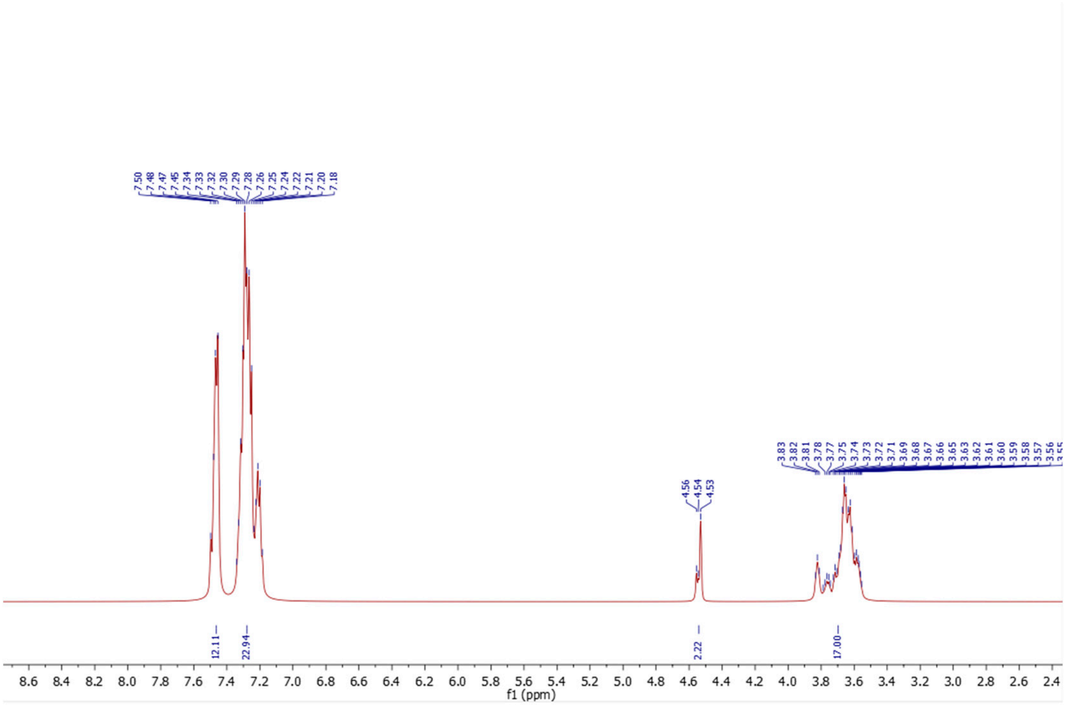

Figure S15. ^{1}H-NMR spectrum for **40** ($CDCl_3$)

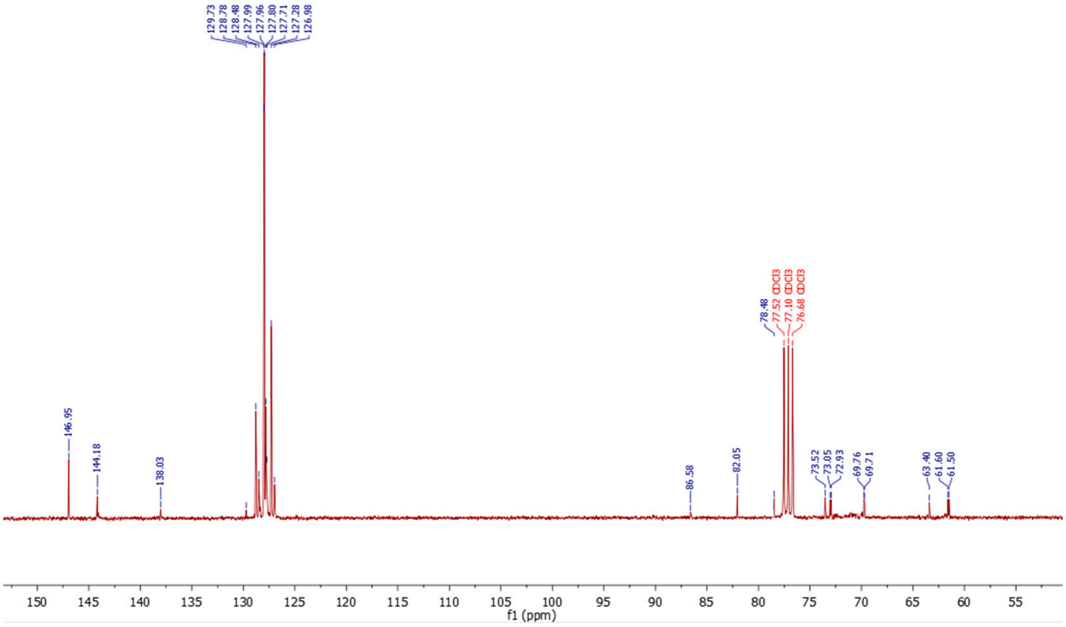

Figure S16. ^{13}C-NMR spectrum for **40** ($CDCl_3$)

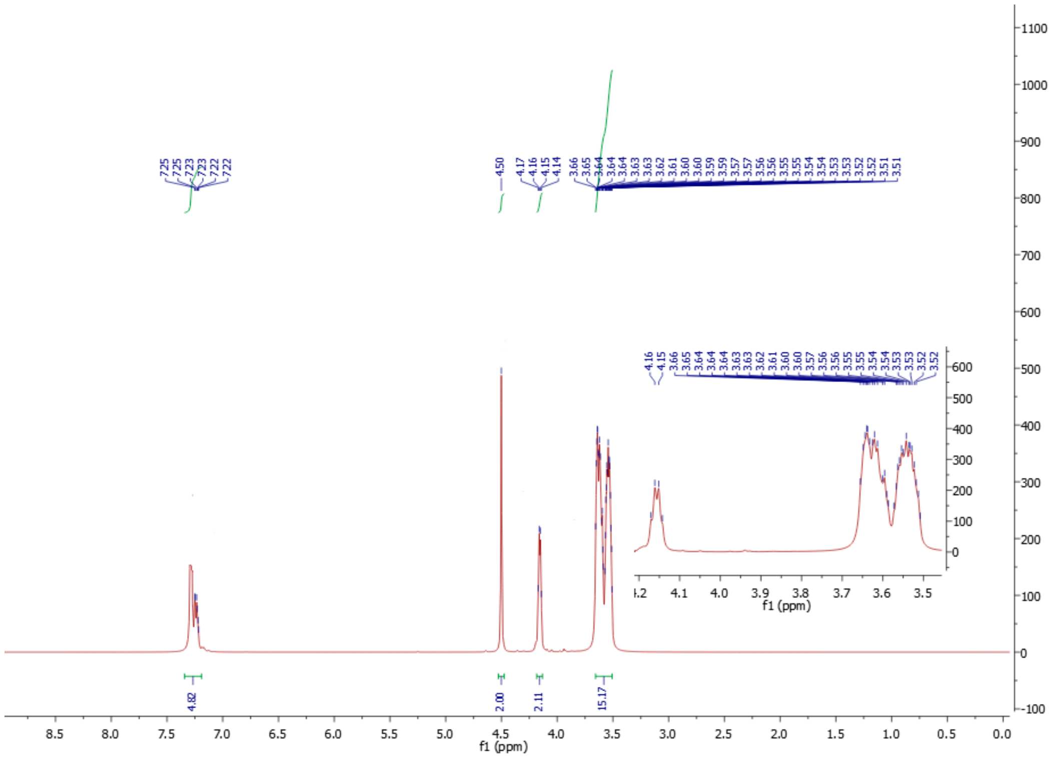

Figure S17. ^{1}H-NMR spectrum for **41** ($CDCl_3$ + D_2O)

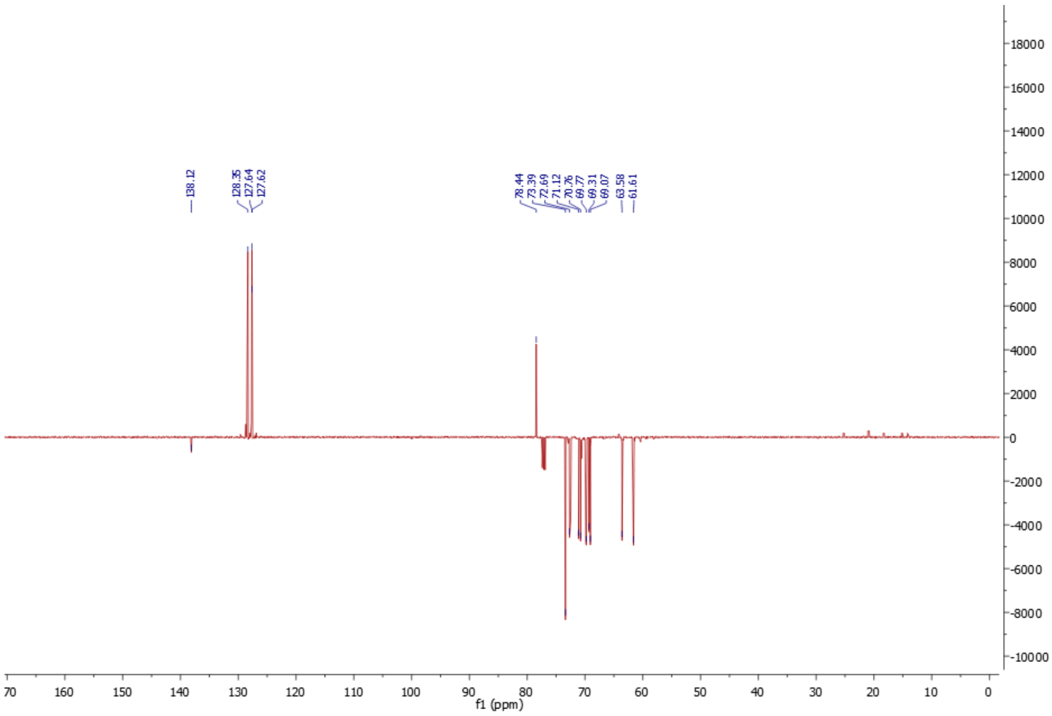

Figure S18. ^{13}C-NMR spectrum for **41** ($CDCl_3$)

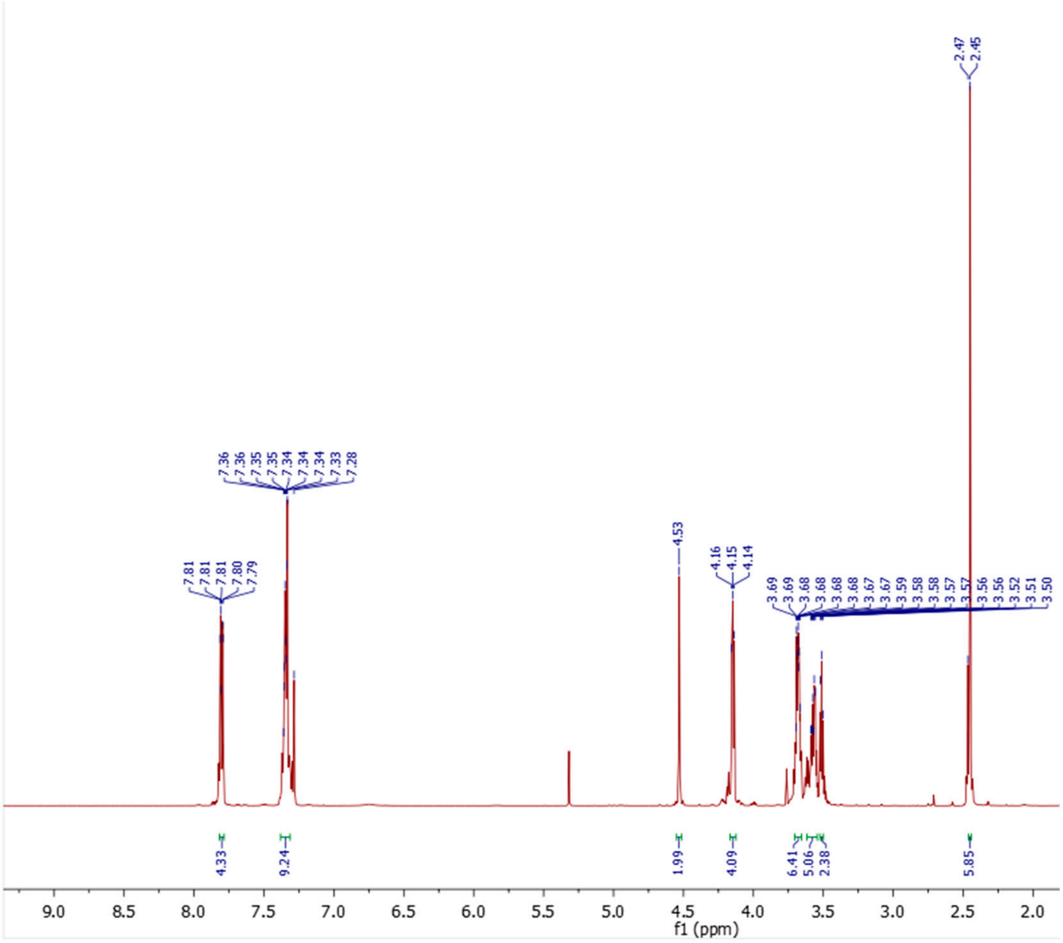

Figure S19. ^{1}H-NMR spectrum for **42** ($CDCl_3$)

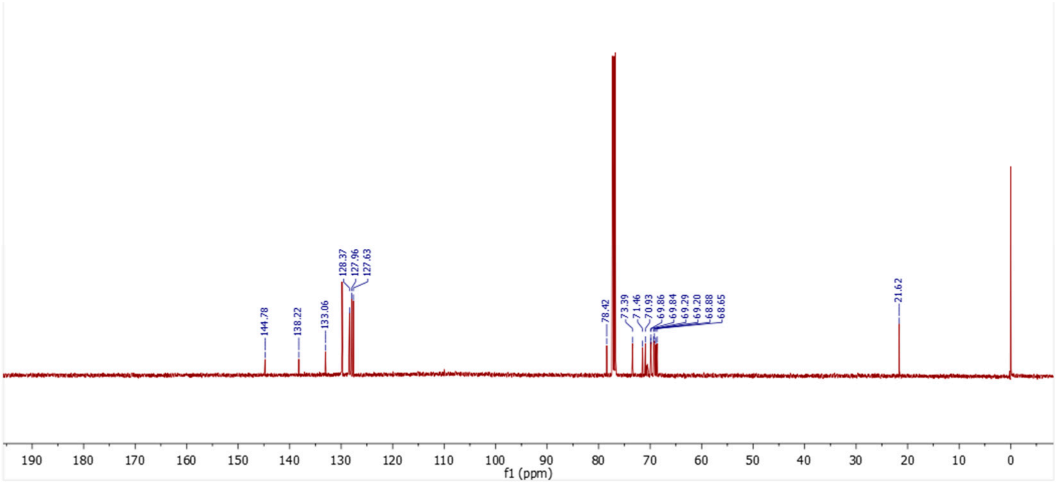

Figure S20. ^{13}C-NMR spectrum for **42** ($CDCl_3$)

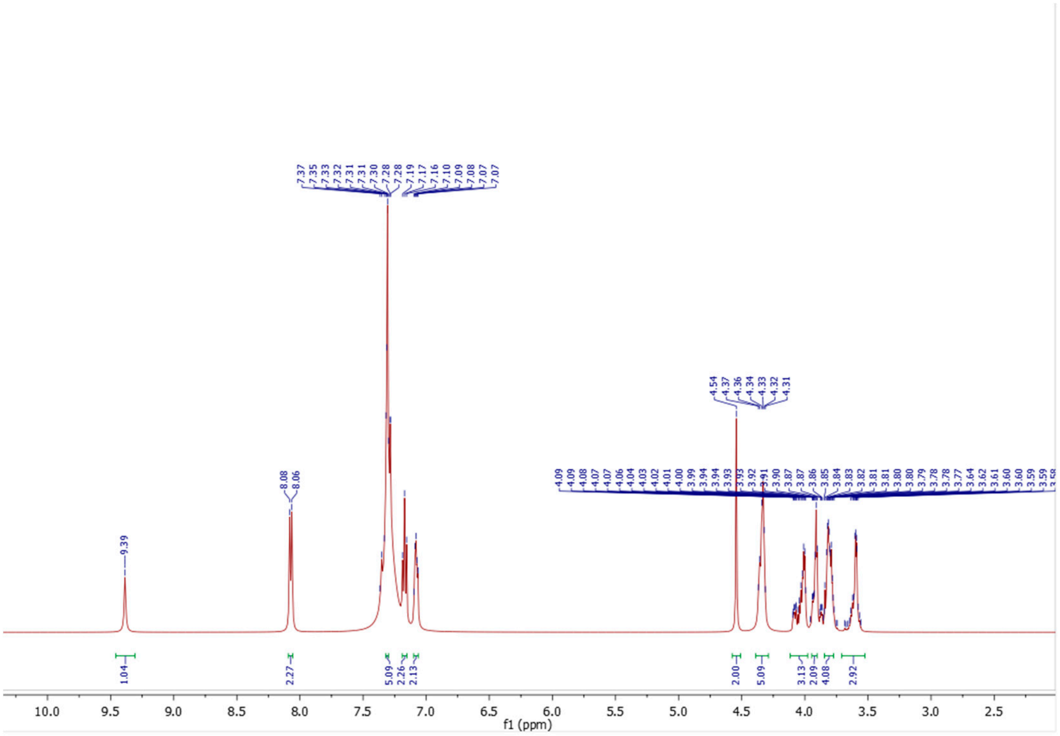

Figure S21. ^{1}H-NMR spectrum for **43** ($CDCl_3$)

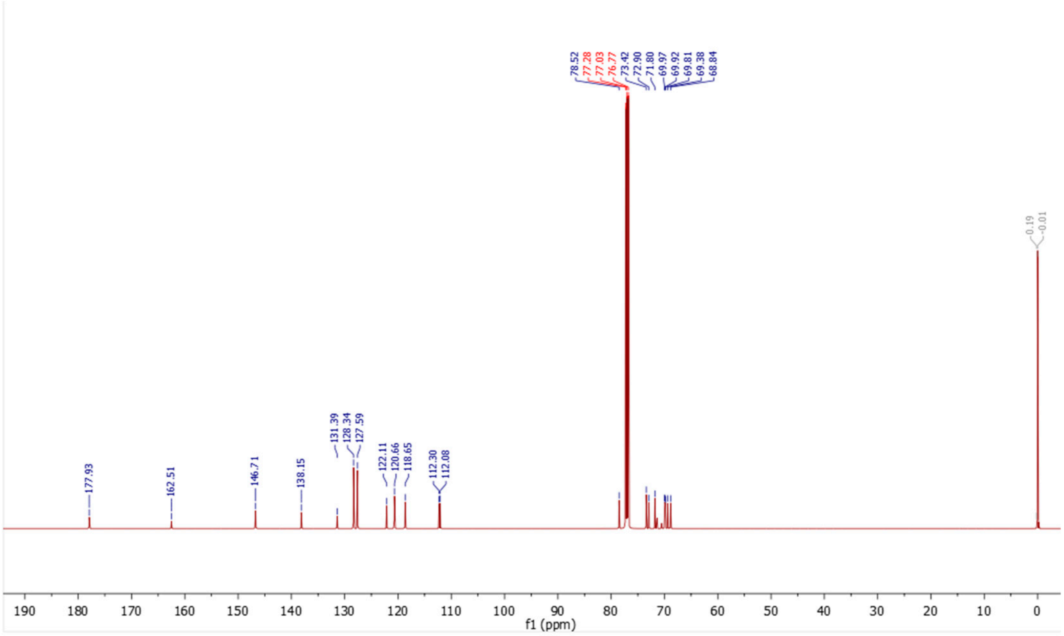

Figure S22. ^{13}C-NMR spectrum for **43** ($CDCl_3$)

2. Investigated ions in selectivity studies

The synthesized new crown ethers were studied by UV-Vis spectroscopy to investigate their complexation properties toward 24 different metal ions (Pb^{2+}, Li^{+}, Na^{+}, K^{+}, Rb^{+}, Ag^{+}, Cs^{+}, Mg^{2+}, Ca^{2+}, Mn^{2+}, Fe^{2+}, Fe^{3+}, Co^{2+}, Ni^{2+}, Cu^{2+}, Zn^{2+}, Sr^{2+}, Pd^{2+}, Cd^{2+}, Ba^{2+}, Hg^{2+}, Al^{3+}, Bi^{3+}, Cr^{3+}) compared to the Pb^{2+}-selective parent compound.

For appropriate water solubility, various counterions were present:

- carbonate counterion: Rb^{2+}, Li^{+}, Cs^{+}
- sulfate counterion: Mn^{2+}, Fe^{2+}
- hydroxide counterion: Ba^{2+}
- chloride counterion: Sr^{2+}, Al^{3+}, Hg^{2+}, Bi^{3+}, Fe^{3+}
- iodide counterion: Cd^{2+}
- acetate counterion: K^{+}, Ni^{2+}, Co^{2+}, Na^{+}, Cu^{2+}, Ag^{+}, Ca^{2+}, Zn^{2+}, Mg^{2+}
- nitrate counterion: Pb^{2+}, Cr^{3+}, Pd^{2+}.

The coordination of these counterions did not take place.
